# Nine residues in HLA-DQ molecules determine with susceptibility and resistance to type 1 diabetes among young children in Sweden

**DOI:** 10.1038/s41598-021-86229-8

**Published:** 2021-04-23

**Authors:** Lue Ping Zhao, George K. Papadopoulos, Antonis K. Moustakas, George P. Bondinas, Annelie Carlsson, Helena Elding Larsson, Johnny Ludvigsson, Claude Marcus, Martina Persson, Ulf Samuelsson, Ruihan Wang, Chul-Woo Pyo, Daniel E. Geraghty, Åke Lernmark

**Affiliations:** 1 Public Health Sciences Division, Fred Hutchinson Cancer Research Center, 1100 Fairview Ave NE, Seattle, WA 98109 USA; 2 Laboratory of Biophysics, Biochemistry, Biomaterials and Bioprocessing, Department of Agriculture, Faculty of Agricultural Technology, University of Ioannina, Ioannina, Greece; 3 Department of Food Science and Technology, Faculty of Environmental Sciences, Ionian University, GR26100 Argostoli, Cephalonia Greece; 4 Department of Pediatrics, Lund University, Lund, Sweden; 5 Department of Clinical Sciences, Lund University CRC, Skåne University Hospital, Malmö, Sweden; 6 Department of Clinical and Experimental Medicine, Crown Princess Victoria Children´S Hospital, Region Östergötland and Div of Pediatrics, Linköping University, Linköping, Sweden; 7 Department of Clinical Science and Education Karolinska Institutet and Institution of Medicine, Clinical Epidemiology, Karolinska Institutet, Stockholm, Sweden; 8 Department of Medicine, Clinical Epidemiological Unit, Karolinska Institutet, Stockholm, Sweden; 9 Clinical Research Division, Fred Hutchinson Cancer Research Center, Seattle, WA USA

**Keywords:** Genetics, Immunology, Diseases, Endocrinology, Medical research

## Abstract

HLA-DQ molecules account over 50% genetic risk of type 1 diabetes (T1D), but little is known about associated residues. Through next generation targeted sequencing technology and deep learning of DQ residue sequences, the aim was to uncover critical residues and their motifs associated with T1D. Our analysis uncovered (αa1, α44, α157, α196) and (β9, β30, β57, β70, β135) on the HLA-DQ molecule. Their motifs captured all known susceptibility and resistant T1D associations. Three motifs, “DCAA-YSARD” (OR = 2.10, p = 1.96*10^−20^), “DQAA-YYARD” (OR = 3.34, 2.69*10^−72^) and “DQDA-YYARD” (OR = 3.71, 1.53*10^−6^) corresponding to DQ2.5 and DQ8.1 (the latter two motifs) associated with susceptibility. Ten motifs were significantly associated with resistance to T1D. Collectively, homozygous DQ risk motifs accounted for 43% of DQ-T1D risk, while homozygous DQ resistant motifs accounted for 25% protection to DQ-T1D risk. Of the identified nine residues five were within or near anchoring pockets of the antigenic peptide (α44, β9, β30, β57 and β70), one was the N-terminal of the alpha chain (αa1), one in the CD4-binding region (β135), one in the putative cognate TCR-induced αβ homodimerization process (α157), and one in the intra-membrane domain of the alpha chain (α196). Finding these critical residues should allow investigations of fundamental properties of host immunity that underlie tolerance to self and organ-specific autoimmunity.

## Introduction

Type 1 diabetes (T1D) is an autoimmune disease, due to the destruction of normal pancreatic islet beta-cells by the host immune system, and accounts for the majority of all diabetes among young children^1,2^. Further, earlier twin and family studies underline the major role of genetic factors together with environmental factors^1–4^. Using modern genotyping technologies, recent genome wide association studies (GWAS) have uncovered ~ 50 single nucleotide polymorphisms (SNPs) in multiple non-HLA immune genes explaining probably less than 25% T1D associations^5–7^. In contrast, class II genes in the human leukocyte antigen (HLA) system have been researched for several decades, and strongly contribute to the genetic association with T1D, accounting for ~ 50% of genetic association with T1D^8,9^. Class II genes include the multiallelic HLA-DR*A1-B1 haplotype coding for the DRαβ heterodimer (α-chain essentially monomorphic), and the HLA-DQ*A1-B1 haplotype coding the DQαβ heterodimer. Due to their genetic proximity, *HLA-DR* and *-DQ* genes are in linkage disequilibrium (LD), and thus empirically separating their individual associations with T1D is challenging^9–11^. Conventionally, DQ molecules exhibit stronger empirical associations with T1D. Our recent effort focused on identification of specific DQ risk residues among subjects with high-risk HLA-DQ haplotypes (DQ2.5 and DQ8.1)^12^ as well as those resistant residues among subjects with low risk HLA-DQ haplotypes^13^, complementing earlier works on HLA-DR molecules^14,15^.

Integrating susceptible and resistant DQ residues, the current investigation was to explore their genotypic associations with T1D, especially, assessing heterodimeric associations with DQA1 and DQB1 alleles on the same chromosome (cis) and between homologous chromosomes (trans), with specific focus on the DQ2.5 and DQ8.1 haplotypes. Furthermore, we assessed if DR3 and DR4, two major risk alleles of HLA-DRB1, confounded HLA-DQ associations with T1D through haplotypic association analysis. To gain insight into autoimmunity, we centered our analysis on DQ motif associations with six biochemically defined islet autoantibodies measured at the time of T1D diagnosis^16^. To replicate discovered associations, we utilized a case–control study from the Type 1 Diabetes Genetic Consortium (T1DGC) to carry out T1D associations with discovered motifs^17^. Last, in a final analysis we estimated how much susceptibility and resistant DQ motifs contributed to either the excess or reduction of risk for T1D.

## Results

### Critical residues among subjects with risk or resistant DQ haplotypes

**Figure 1:**
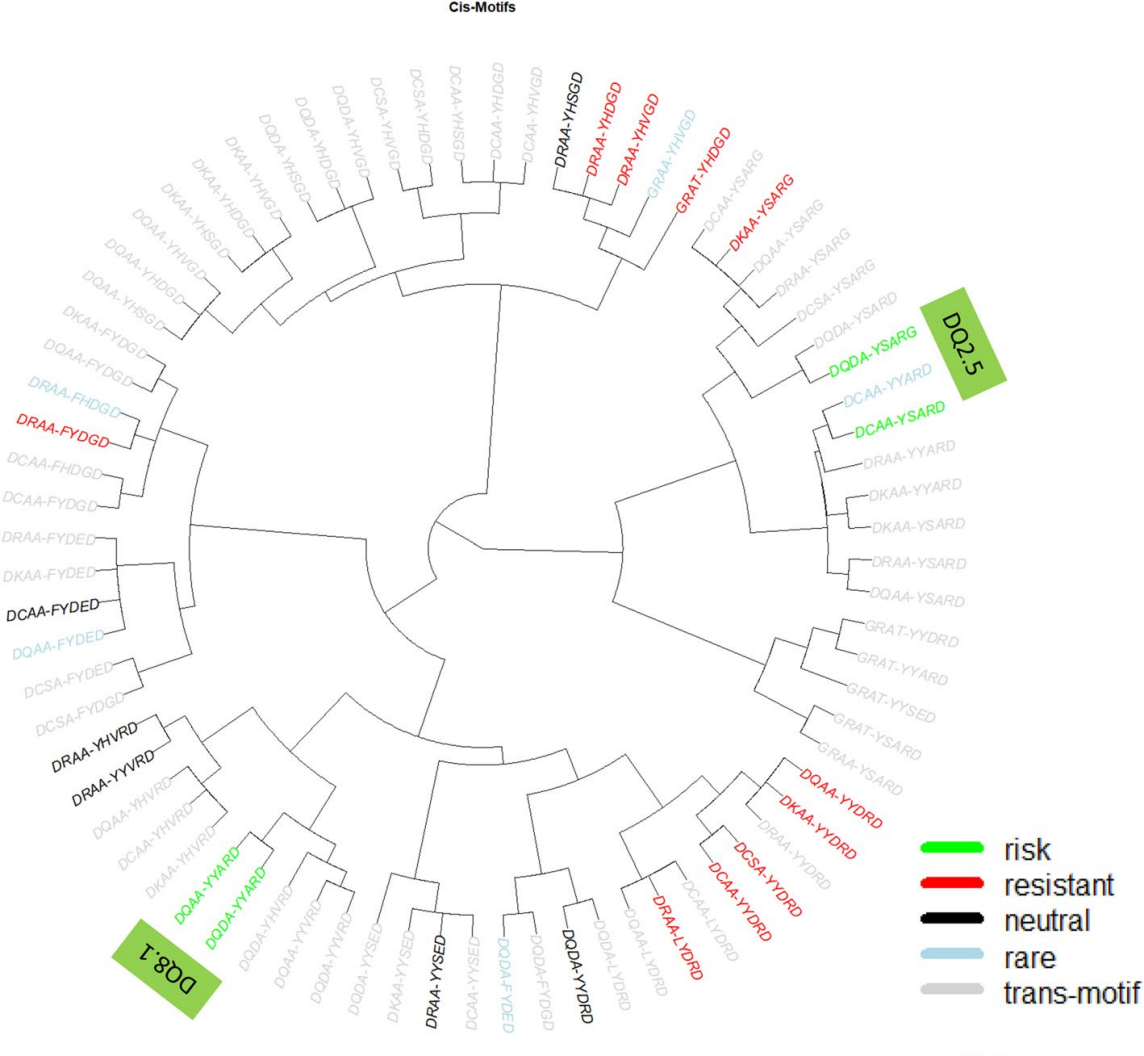
Phylogenic representation of all cis DQ motifs that are risk to (p < 0.05 and OR > 1, colored green), resistant to (p < 0.05, OR < 1, colored red), neutral (p > 0.05, black), and rare (with fewer than 5 observed motif copies, colored blue), together with all trans-DQ motifs (colored gray).

*DQA1* and *DQB1*, which are in high LD and their alleles mostly form cis-haplotypes, are translated to form respectively the alpha and beta chain of the heterodimeric HLA-DQ molecule. DQ haplotypes were polymorphic and had 45 unique haplotypes in our case–control study, each of which consisted of unique residues (complete lists of amino acid sequences of mature molecules in Supplementary Figs. S1 and S2). Our phylogenic analysis of sequence similarities showed that these DQ haplotypes were hierarchically organized into seven haplotype clusters; 1 to 7 (Fig. 1). Clusters 2 and 4 included susceptible motifs DQ2.5 and DQ8.1 (motifs colored green), and carriers of all DQ motifs in these two clusters were at high T1D risk. Among high-risk subjects, hierarchically organized haplotype (HOH) analysis identified three critical residues (α44, β57, β135) that captured disease association with HLA-DQ among subjects with high-risk DQ haplotypes^12^. The remaining clusters included largely resistant DQ haplotypes, and the HOH analysis of subjects revealed seven critical residues (αa1, α156, α196, β9, β30, β57, β70) that accounted for autoimmunity in T1D^13^.

### Four alpha and five beta residues were critically associated with T1D risk

Integrating all risk and resistant residues in the DQ molecule led to four alpha residues (αa1, α44, α157, α196) and five beta residues (β9, β30, β57, β70, β135) that were critically associated with either susceptibility or resistance to T1D. To assess roles of these residues in T1D associations, we investigated residue-specific frequencies of corresponding amino acids between controls and patients, who had high and low DQ risk haplotypes (Table 1). Residue α44 took four possible amino acids (C, K, Q, R); α44C and Q were strongly associated with high T1D risks (OR = 2.10 and 3.32, p = 1.96*10^−20^ and 1.00*10^−86^, respectively), α44K was strongly associated with resistance to T1D risk (OR = 0.27, p = 1.85)^13^ and α44R was absent among high-risk DQ subjects. Among low risk DQ subjects, the resistant α44C and Q had significant associations with resistance to T1D risk (OR = 0.39 and 0.6, p = 5.42*10^−19^ and 2.59*10^−3^, respectively), and the residue α44R was highly associated with the resistance to T1D (OR = 0.40 and p-value = 5.20*10^−59^). Interestingly, the amino acid α44K was absent in low risk subjects. When contrasting the associations of α44C and Q between high and low risk subjects, their differences of risk and resistant associations were highly significant (exact Fisher’s p-value = 2.16*10^−31^ and 1.92*10^−19^, respectively). On the other hand, the beta chain residues β57A (OR = 2.31; p = 5.46*10^−99^) and D (OR = 0.46; 3.93*10^−4^) had reverse associations with T1D risk among high-risk subjects. Among the low risk subjects, the first residue, β57A, had no significant association to T1D risk (OR = 0.93, p = 0.89), while the second residue, β57D, was associated with resistance to T1D (OR = 0.25, p = 2.23*10^−96^). Two amino acids, β57S/V, were absent among high-risk subjects and had no associations (p = 0.11 and 0.78, respectively). Last, β135D and G had opposite associations with T1D high-risk subjects (OR = 2.47 and 0.40, p = 6.06*10^−104^ and 2.00*10^−7^, respectively). Among the low risk subjects, β135D had significant resistance to T1D (OR = 0.41, p = 1.84*10^−91^), while β135G was absent. The disease associations with residue β135D were completely opposite to each other between high and low risk subjects (Fisher’s exact test; p = 2.88*10^−117^).

**Table 1:**
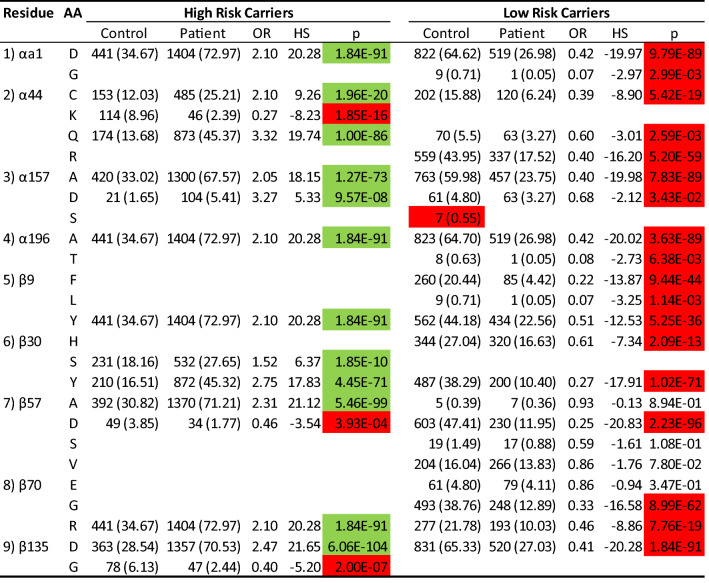
Results from association analysis of nine selected residues (αa1, α44, α157, α196, β9, β930, β57, β70, β135) with T1D in the case-control study with 636 control and 962 patients with estimated allelic frequencies (%), odds ratio, hap-score and p-value among carriers of DQ2.5 and DQ8.1 and non-carriers.

From the perspective of resistant residues, αa1D and G among low risk subjects were significantly associated with resistance to T1D (OR = 0.42 and 0.07, p = 9.79*10^−89^ and 2.99*10^−3^, respectively). Among high-risk subjects, residue αa1D had significant association with susceptibility to T1D (OR = 2.10, p = 1.84*10^−91^), while αa1G was absent. The association with αa1D was significantly different between low and high-risk subjects (exact Fisher’s p = 7.87*10^−102^). The next residue α157 took three possible amino acids (A, D, S); the first two, α157A/D, had significant resistance associations (OR = 0.40 and 0.68, p = 7.83*10^−89^ and 0.034, respectively), while α157S was absent among patients but had seven allelic copies among controls with low risk DQ haplotypes. Among high-risk subjects, α157A/D were significantly associated with susceptibility to T1D (OR = 2.05 and 3.27, p = 1.27*10^−73^ and 9.57*10^−8^, respectively), while the third residue, α157S, was absent. Again, T1D risk associations with α157A/D were significantly different between low- and high-risk subjects (exact Fisher’s p = 9.18*10^−97^ and 4.93*10^−8^, respectively). The next residue, β9, had three amino acids associated with resistance (F, L, Y; OR = 0.22, 0.07 and 0.51, p = 9.44*10^−44^, 1.14*10^−3^ and 5.25*10^−36^, respectively) among low-risk subjects. Among high-risk subjects, β9F and L were absent, while β9Y was associated with T1D risk (OR = 2.10, p = 1.84*10^−91^), and the association was significantly different between high and low risk (exact Fisher’s p = 3.74*10^−66^). Among low-risk subjects, residue β30 had two resistant amino acids β30H/Y (OR = 0.61 and 0.27, p = 2.09*10^−13^ and 1.02*10^−71^, respectively), and β30S was not observed in such carriers. Among high-risk subjects, β30S/Y had significant associations with susceptibility to T1D (OR = 1.52 and 2.75, p = 1.85*10^−10^ and 4.45*10^−71^, respectively), but β30H was not observed in these subjects. For amino acid β30Y, its association with T1D was significantly different between low- and high-risk subjects (Fisher’s exact test; p = 7.62*10^−106^). The ninth residue, β70, had two amino acids significantly associated with resistance, G and R (OR = 0.33 and 0.46, p = 7.76*10^−19^ and 1.84*10^−91^, respectively), and had one amino acid, β70E, with a neutral association (OR = 0.86, p = 0.34) among low-risk subjects. No high-risk subjects were found with β70E or G, while β70R was significantly associated with T1D (OR = 2.10, p = 1.84*10^−91^), and its association was significantly different between high- and low-risk subjects (exact Fisher’s p = 2.19*10^−45^).

In summary, all these nine residues had highly significant associations with T1D among subjects with either low- or high-risk DQ haplotypes. Strikingly, the same amino acids could exhibit paradoxical associations between low- and high-risk subjects. Further, some of common amino acids in the high-risk group are absent from the low-risk group, and vice-versa. Note that some motifs listed in Table 2 had fewer than 10 observed copies, e.g., ID 13, 18, 19 and 20, and their associated p-values were not robust enough and should be taken as suggestive values.

**Table 2:**
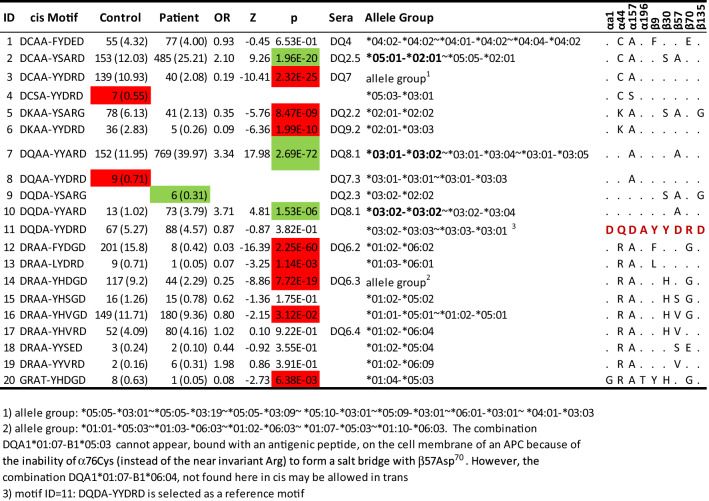
Allelic association analysis of type 1 diabetes with DQA1-DQB1 motifs of (αa1, α44, α157, α196, β9, β30, β57, β70, β135): estimated frequency (%) among control and patient, estimated odds ratios, Z-score, and p-value, corresponding serotype, and equivalent allelic groups, in addition to profiles of amino acids across all motifs

Five rare motifs, with fewer five observed motif copies, are excluded. In total we have 1266 and 1921 motif copies observed in 636 controls and 962 patients, respectively, after excluding 6 and 3 motif copies from respective groups.

### Motifs of nine residues account for DQ susceptibility and resistance to type 1 diabetes risks

Four alpha residues and five beta residues originated from transcription in cis and haplotypes of these nine amino acids are referred to as cis DQ motifs. In total, there were 25 unique DQ motifs: four susceptibility motifs (highlighted in green), ten resistance motifs (highlighted in red), and six neutral motifs with insignificant associations (p-values > 0.05) (Table 2). Five rare motifs with fewer than five observations were shown only in the Supplementary Table S1. The motif “DCAA-YSARD”, corresponding to DQA1*05:01-B1*02:01 and A1*05:05-B1*02:01, was significantly associated with susceptibility to T1D (OR = 2.10, p = 1.96*10^−20^), and is equivalent to the traditionally known DQ2.5 serotype. The motifs “DQAA-YYARD” and “DQDA-YYARD”, corresponding to traditionally known DQ8.1 serotypes, had significant association with T1D susceptibility (OR = 3.34 and 3.71, p = 2.69*10^−72^ and 1.53*10^−6^, respectively). Last, the susceptible motif “DQDA-YSARG” was absent among controls and had six copies with allelic frequency of 0.31% among patients.

Among twenty HLA-DQ motifs, ten were associated with resistance to T1D. The motif “DRAA-FYDGD”, uniquely corresponding to the well-known protective DQ6.2 haplotype, conveyed significant resistance to T1D risk (OR = 0.03, p = 2.25*10^−60^). The next resistance motif “DCAA-YYDRD”, motif 3, corresponding to a group of HLA-DQ haplotypes listed in the footnote, was significantly resistant to T1D (OR = 0.19, p = 2.32*10^−25^). The third most protective motif, “DRAA-YHDGD”, was associated with resistance to T1D (OR = 0.25, p = 7.72*10^−19^). The other five protective motifs were “DKAA-YSARG”, “DKAA-YYDRD”, “DRAA-LYDRD”, “DRAA-YHVGD” and “GRAT-YHDGD”, with p-values less than 0.05. Two motifs, “DCSA-YYDRD” and “DQAA-YYDRD”, were observed (with more than five copies) only among controls (Table 2).

Given the substantially different genetic distributions of residues between low- and high-risk subjects (Table 1), we assessed motif associations stratified by low or high DQ risks. As expected, distributions of DQ motifs were rather distinct between subjects with low and high DQ risks (Table S1). The most of commonly observed motifs were specific to subjects with high or low risk DQ haplotypes, and their associations with T1D were largely consistent with those in the combined low and high-risk subjects. For example, the motif “DCAA-YSARD” was observed only among high-risk subjects, and “DCAA-YYDRD” was observed only among low risk subjects. Hence, in all combined data, their respective association with susceptibility or resistance for T1D were largely unchanged. One exception, to this general observation, was the motif “DQDA-YYARD”, which was significantly associated with susceptibility to T1D risk among high-risk subjects (OR = 4.56, p = 4.30*10^−7^) but was relatively uncommon among low risk subjects and showed no indication of associations with T1D risks (OR = 0.88, p = 0.87).

### Subtle variations of residues alter association profiles with T1D

The directly constructed profile of disease-associated motifs permitted us to examine how subtle variations of specific residues may alter their T1D associations (right panel of Table 2). The motif “DCAA-YSARD”, corresponding to DQ2.5, differed from the adjacent motif “DCAA-YYDRD” with only two residues at β30 and β57, but their T1D associations were completely opposite and reverted from susceptibility to resistance (OR = 2.10 and 0.19, p = 1.96*10^−20^ and 2.32*10^−25^, respectively), the difference between the two was significant (Fisher’s extact test; p = 4.24*10^−39^). The next motif pair “DQAA-YYARD”, corresponding to DQ8.1, and “DKAA-YYDRD”, differed only at α44 and β57, and their T1D associations were opposite (OR = 3.34 and 0.09, p = 2.69*10^−72^ and 1.99*10^−10^, respectively), the difference was significant (Fisher’s exact test; p = 5.04*10^−22^). The next DQ8.1 motif “DQDA-YYARD” differed from the adjacent motif “DQDA-YYDRD” only at residue β57, but the former was associated with susceptibility to T1D (OR = 3.71, p = 1.53*10^−6^) and the other was neutral (OR = 0.87, p = 0.38). The difference of the associations between these two motifs was significant (Fisher’s extact test; p = 7.48*10^−6^). It was also of interest to contrast the uncommon motif 8, “DQAA-YYDRD”, with motif 7, “DQAA-YYARD”, and they were nearly identical except for β57. In fact, all five alleles in both motifs had an identical alpha chain, DQA1*03:01. Their associations differed significantly from each other (Fisher’s exact test; p = 1.16*10^−7^).

### Genotypes of DQ motifs have profound associations with T1D

Each subject carries a pair of DQ motifs, i.e., a motif genotype, and two motifs were jointly associated with T1D. The combination of 20 motifs (Tables 2 and S2) resulted in several rare genotypes with fewer than 5 observations. Upon merging of all rare genotypes into a single category, there was a total of 60 distinct genotypes (Table S2), and 25 were deemed to have neutral associations with corresponding p-values greater than 0.05. The genotype association results for 35 genotypes were sorted by Z-scores (Table 3). The first eight genotypes were significantly associated with susceptibility to T1D, with OR ranging from 2.46 to 14.54 (p-values ranged from 0.012 to 4.14*10^−21^). Next thirteen genotypes were significantly associated with resistance to T1D risk with OR ranging from 0.07 to 0.30 (p-values ranged from 0.038 to 3.69*10^−9^). The last fourteen genotypes, which had frequencies of five or more copies were observed among controls, and none were among patients, and were thus deemed to be highly resistant, perhaps protective, to T1D risks.

**Table 3:**
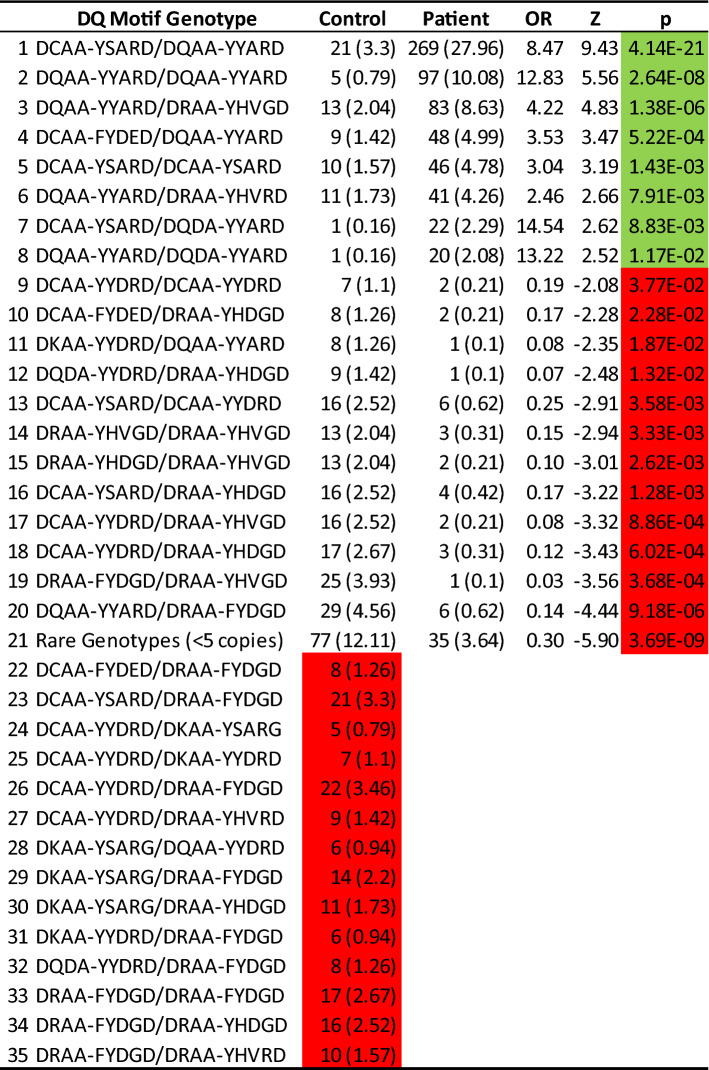
Genotypic association analysis of type 1 diabetes with DQA1-DQB1 motifs of (αa1, α44, α157, α196, β9, β30, β57, β70, β135): estimated frequency (%) among control and patient, estimated odds ratios, Z-score, and p-value, corresponding serotype, and equivalent allelic groups.

Listed are genotypes with p-values less than 0.05. Genotypic association results for all genotypes are listed in Table S2. The logistic regression analysis used the offset to compute odds ratio with the "virtual genotype" under the null as the reference.

### DQ2.5 and DQ8.1 genotypic effects

Out of 20 common DQ motifs, three motifs “DCAA-YSARD”, “DQAA-YYARD” and “DQDA-YYARD”, and the relatively uncommon “DQDA-YSARG”, were significantly associated with susceptibility to T1D. Given their susceptibilities, it was important to investigate specific genotype associations of these three DQ motifs, with themselves (homozygotes), with each other (doubly heterozygote), with neutral motifs (showing motif-specific effect), and with resistant motifs (showing additive motif effect); neutral motifs included all motifs with associated p-values greater than 0.05 and resistant motifs with p-values less than 0.05 and odds ratios less than 1. We conducted a univariate association analysis to compare each genotype compared to all other genotypes combined through a logistic regression model, estimating coefficient, odds ratio, standard error, Z score and p-value (Table 4).

**Table 4:**
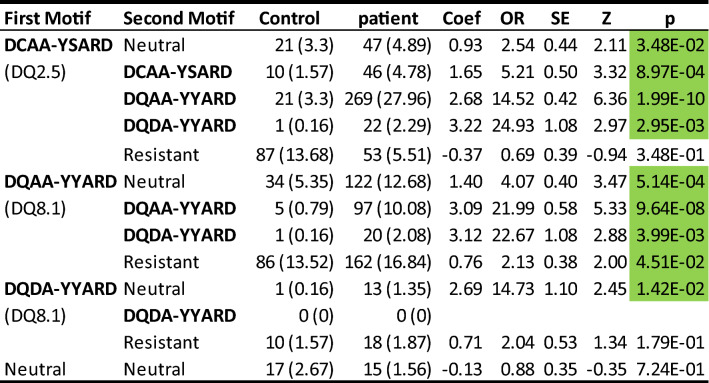
Results from the univariate association analysis for three high risk DQ motifs (DQ2.5: DCAA-YSARD, DQ8.1: DQAA-YYARD and DQ8.1: DQDA-YYARD), after accounting for all neutral and resistant motifs.

Numbers and frequence percentages of genotype groups are listed for controls and patients, and so are estimated coef (log odds ratio), standard error, Z score, and p-value. The reference genotype is the specific genotype versus all others.

Pairing the motif “DCAA-YSARD” (DQ2.5) with neutral motifs, itself, two DQ8.1 motifs and resistant motifs resulted in five possible genotypes (Table 4). The heterozygote with neutral motifs showed a significant association with susceptibility to T1D (OR = 2.54, p = 0.035), which was largely due to the motif “DCAA-YSARD”. As expected, its homozygote had a significant association with susceptibility to T1D with OR = 5.21 (p = 8.97*10^−4^), with an effect that was nearly doubled that of the heterozygote with neutral motifs (OR = 2.54). The heterozygote “DCAA-YSARD” (DQ2.5)/ “DQAA-YYARD” (DQ8.1) was associated with markedly increased susceptibility to T1D (OR = 14.52, p = 1.99*10^−10^). Similarly, the heterozygote “DCAA-YSARD” (DQ2.5)/ “DQDA-YYARD” (DQ8.1) was associated with an even greater susceptibility (OR = 24.93, p = 2.95*10^−3^), although it had a lower genotype frequency. Last, the heterozygote with resistant motifs had insignificant association with T1D (OR = 0.69, p = 0.35), probably due to additive effect from both susceptibility and resistance motifs.

Pairing the motif “DQAA-YYARD” with neutral motifs, itself, “DQDA-YYARD” and resistant motifs, showed similar effects, albeit with some differences. The heterozygote with the neutral motifs captured the pure contribution of the motif “DQAA-YYARD” to T1D, with a significant association with susceptibility to T1D (OR = 4.07, p = 5.14*10^−4^). The homozygote had much elevated association with T1D susceptibility (OR = 21.99, p = 9.64*10^−9^). The heterozygote with another DQ8.1 “DQDA-YYARD” was also highly associated with increased susceptibility (OR = 22.67, p = 3.99*10^−3^). Last, the heterozygote with resistant motifs had much weaker association (OR = 2.13, p = 0.014), because resistant motifs compromised some of disease associations but not to the same extent (ORs from 0.03 to 0.35, Table 2).

Examining in the same manner another DQ8.1 motif, motif 10, i.e. “DQDA-YYARD”, its heterozygote with neutral motifs had significant association with T1D susceptibility (OR = 14.73, p = 0.0142), which captured its sole contribution. There were no homozygotes in this relatively large study population, since the expected genotype frequency is in the order of 1.44*10^−3^. The heterozygote with resistant motifs showed insignificant association with T1D risk (OR = 2.04, p = 0.18), probably due to relatively low genotypic frequency.

By estimated odds ratios, the heterozygote “DCAA-YSARD/DQAA-YYARD” had an OR of 14.52, which was slightly greater than the product of individual contributions 10.34 (= 2.54 × 4.07) from “DCAA-YSARD/neutral” and “DQAA-YYARD/neutral”, respectively. Similarly, the heterozygote “DCAA-YSARD/DQDA-YYARD” had an OR of 24.93, which was slightly less than the product of individual contributions 37.41 (= 2.54 × 14.73) from “DCAA-YSARD/neutral” and “DQDA-YYARD/neutral”.

### Expression in trans of DQA1 and DQB1 motifs enriches cellular diversity of DQ heterodimers but has limited associations with T1D

Heterodimeric formation of the DQ molecule with doubly heterozygous DQA1 and DQB1 genotypes could produce four different DQ molecules: two cis-formations with alleles on the same chromosome and two trans-formations across homologous chromosomes, which may not be observed experimentally, as some are structurally prohibited^18^ (see also Fig. 1). For homozygotes or semi-homozygotes, DQA1-B1 motifs on different chromosomes, formed trans-DQ motifs (whenever structurally allowed) that were the same as those cis-DQ motifs. In total, in trans-formation created 73 DQ motifs, of which 48 were new -DQ motifs, several of which were structurally forbidden^18–20^ (see Supplementary Table S3). In a systematic assessment of all trans-motifs, we evaluated potential conditional association of each trans-motif with T1D, after adjusting for all cis-motifs. After excluding trans-motifs with fewer than five copies, we reported conditional associations with 45 trans-motifs. Most of trans-motifs did not exhibit any meaningful adjusted associations, with a few exceptions, e.g., “DQDA-YYARD” (p = 0.01) (see Supplementary Table S3). The many permitted trans-motifs are indicated in the column “Prmt”. In the table, estimated coefficient quantifies adjusted log odds ratio for cis-motifs, together with estimated standard error, Z-score and p-value. Should there be an interest in marginally estimated odds ratio, it was possible to take the ratio of patient and control trans-motif frequencies. For example, the trans-motif 24 “DQAA-YSARD” had the marginal odds ratio of 8.37, but the adjusted odds ratio was 1.26, which was not significantly different from null (p-value = 0.387), because associated cis-motifs explained most of the risk associations. In summary, trans-motifs did not appear to exhibit additional associations, after accounting for all 25 cis-motifs. However, this empirical result from the conditional adjustment did not exclude their potential biological associations between trans DQ motifs and T1D as the cellular expression of DQ heterodimers are enriched.

### DQ associations may be influenced by its linkage-disequilibrium with DRB1

*DQA1* and *DQB1* were genetically nearby *DRB1*, and in high LD with each other, i.e., *DR3* and *DQ2.5* were linked, and so were *DR4* and *DQ8.1*. With DQ motifs of nine residues, we performed haplotype association analysis of DQ motifs and DRB1, with six DR4 subtypes specified by four-digit resolution. Clearly, *DR3* was uniquely linked with the motif “DCAA-YSARD” (DQ2.5), and their joint haplotype had significant association with T1D susceptibility (OR = 2.10, p = 1.96*10^−20^) (Table 5). Similarly, DRB1*04:02, *04:03, *04:04, *04:05 and *04:07 were linked DQ motifs with variable associations. Hence, these empirical haplotype associations prohibited separating DRB1 and DQ associations. However, the same DRB1*04:01 was on the same haplotypes with DQ motifs “DQAA-YYARD”, “DQDA-YYARD”, or “DQDA-YYDRD”. These three haplotypes had variable associations (OR = 5.52, 4.25 and 0.89, p = 4.94*10^−70^, 7.98*10^−5^ and 0.57, respectively), implying that DQ motifs dictated T1D association in the background of DRB1*04:01. DRB1*04:03 reduced T1D susceptibility when in LD with DQ8.1 (OR = 0.12, while for the DQ8.1 alleles collectively OR = 3.34) even though the number of patients and controls with this haplotype was very low. In the case of the DRB1*15 molecule, the OR is identical to the very tightly linked DQA1*01:02-B1*06:02 haplotype. In fact, the number of patients and controls is nearly identical concerning two nearly congruent groups.

**Table 5:**
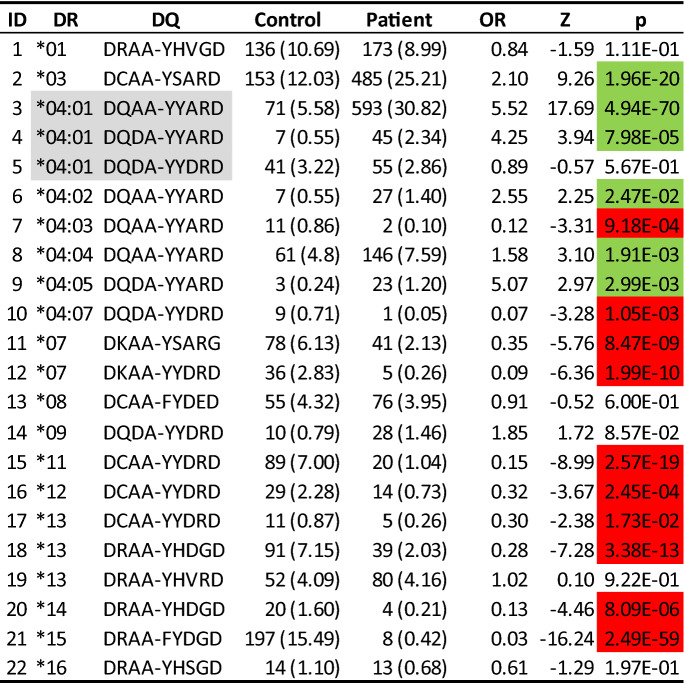
Haplotype association analysis of type 1 diabetes with HLA-DRB1 and DQA1/B1: estimated haplotype frequency (%) among control and patient, estimated odds ratio, Z-score, and p-value, for those haplotypes with five or more copies.

### Motifs of nine residues account for all DQ associations with T1D

**Figure 2:**
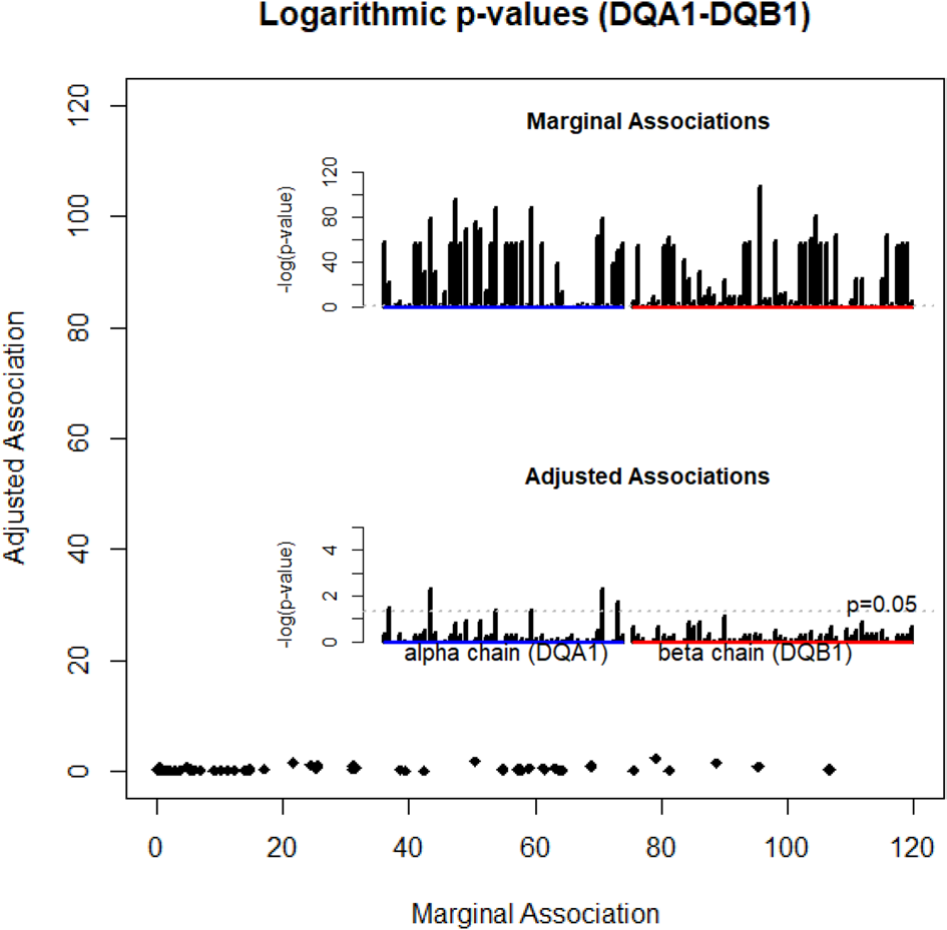
Logarithmic p-values computed for association of type 1 diabetes with the individual residue, with (y-axis) and without (x-axis) adjusting for DQA1-DQB1 motifs.

Besides selected residues on alpha and beta chains, there were 95 polymorphic amino acids across DQ haplotypes in this study population. Due to LD, many residues were expected to have significant marginal associations. We evaluated these marginal associations via a logistic regression of T1D with polymorphic residues. By the log-likelihood ratio test, p-values were computed for each residue, and were shown in the top panel of the bar plot in the Fig. 2. Evidently, many p-values greatly exceeded the threshold of 0.001, and some were approaching 10^−100^ or even less, showing strong marginal associations with T1D. By adjusting for the motifs of selected nine residues, we repeated the same logistic regression analysis, estimating p-values from log-likelihood ratio test (the lower bar plot of the Fig. 2). Indeed, most p-values were insignificant, i.e., p > 0.05, except that several p-values were slightly less than 0.05. We plotted adjusted p-values against marginal p-values as an x–y plot, showing that adjusting motifs accounted for most of the marginal associations (Fig. 2).

### DQ motifs are less diverse among patients than among controls

**Figure 3:**
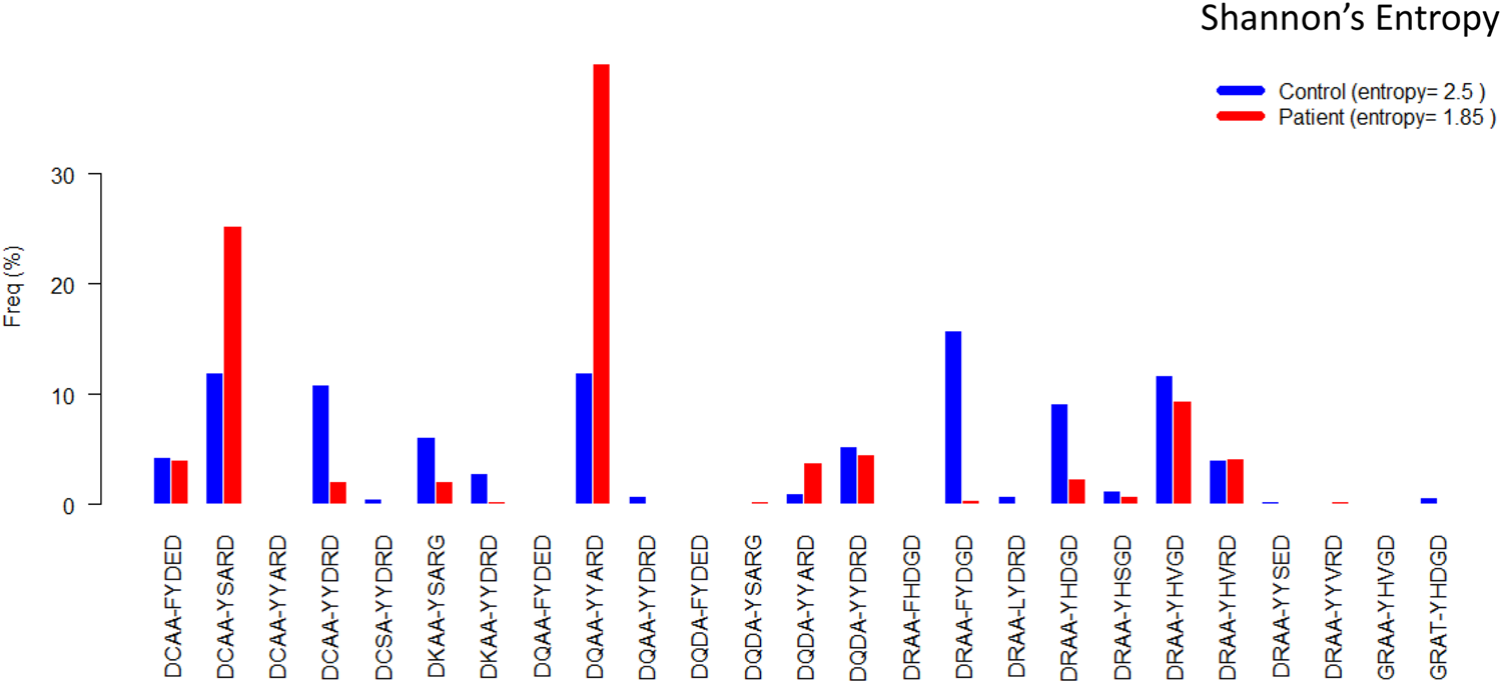
Frequencies of all DQ motifs among 636 controls and 962 patients involved in BDD study.

DQ motifs of selected nine residues retained all susceptibility and resistance to T1D associations that were identified by DQ haplotypes but had reduced polymorphisms due to merging of some DQ haplotypes, especially those rare DQ haplotypes with relatively common ones. The frequencies of 25 DQ motifs among controls and patients (Fig. 3) indicates that the two motifs “DCAA-YSARD” and “DQAA-YYARD”, corresponding to DQ2.5 and DQ8.1, had exceptionally higher frequencies among patients than controls. Further, controls tended to have modest frequencies across many motifs. For this reason, DQ motifs among controls, with a Shannon’s entropy value of 2.50, tended to be more diverse than those among patients with an entropy value of 1.85, where the entropy was used to quantify diversity of genetic polymorphisms^21^.

### Motifs of nine residues capture DQ associations with islet autoantibodies

**Figure 4:**
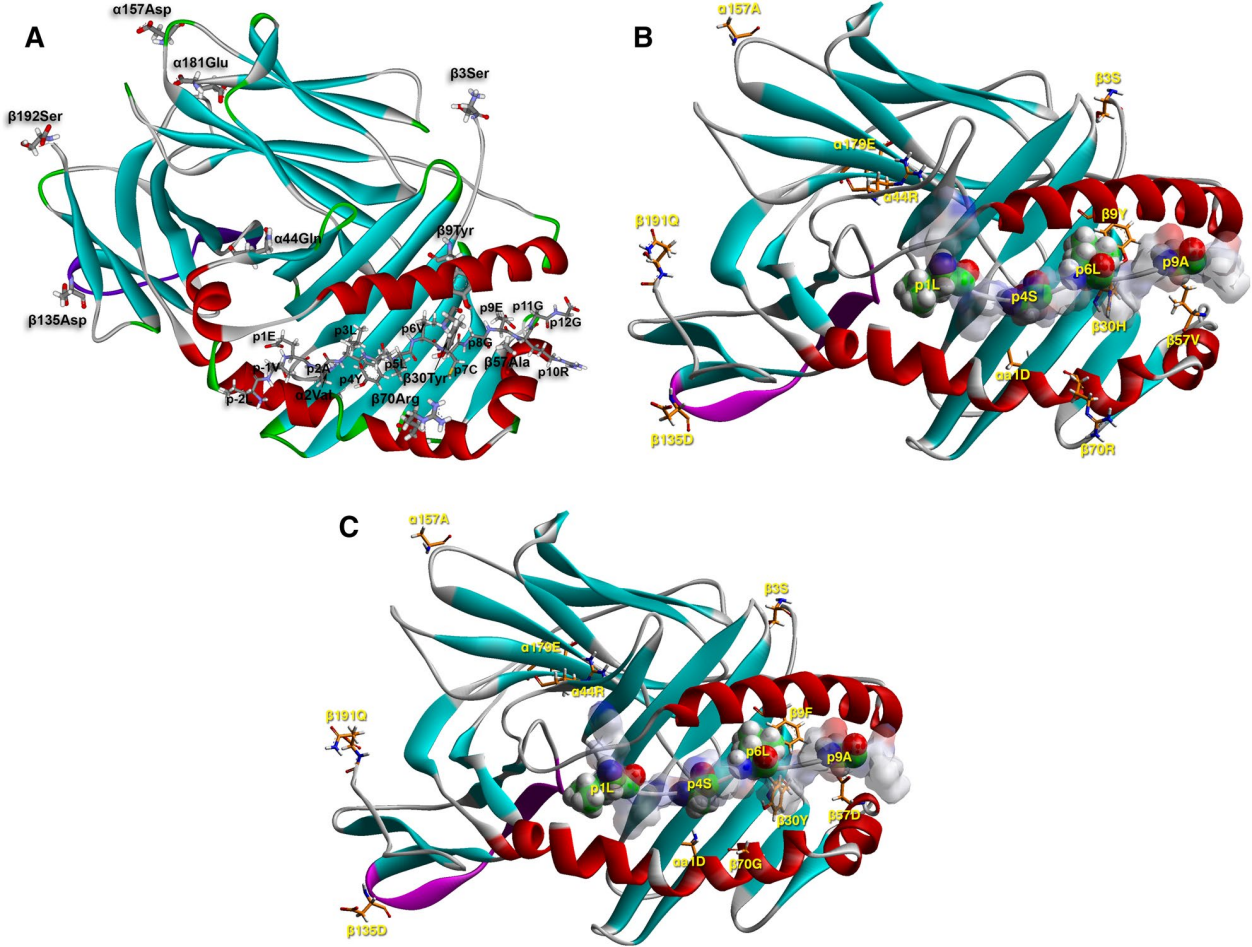
A-C. Molecular depiction of a T1D-susceptible HLA-DQ molecule (DQ8—InsB11-23/B24Gly; 1jk8.pdb, **A.**), a neutral one (DQB1*06:04—InsB5-15, **B.**) and a T1D-resistant molecule (DQB1*06:02—InsB5-15, **C.**) in complex with a so-restricted antigenic peptide from insulin (parts B. and C. obtained upon molecular simulation reported in^23^ based on the crystal structure of the HLA-DQB1*06:02—hypocretin 1–13 complex, 1uvq.pdb^69^. Binding has been demonstrated to the respective epitopes, and in the first case several CD4^+^ T cell clones, specific to this peptide and restricted to HLA-DQ8 have been isolated from patients. The binding register has not been questioned, yet there is evidence that cognate TCRs from T cell clones isolated from patients with T1D recognize an epitope with a shifted register by one residue towards the peptide C-terminus^38^. The HLA-DQ-insulin peptide complexes are depicted in their secondary structure formation (α-helix in red, β-sheet in turquoise, β-turn, random coil or any other form, such as poly-proline II helix of the antigenic peptide backbone, in grey), and the β134-148 CD4-binding stretch in purple. The HLA-DQ AA residues in question are depicted in stick form (atom color convention: carbon, grey; oxygen, red; nitrogen, blue; sulfur, yellow; hydrogen, white). The antigenic peptide is shown in stick form in (**A**) (thinner sticks, with the same color convention), and in space-filling form in (**B**). and (**C**). with the p1Leu, p4Ala, p6Leu and p9Ala anchor residues opaque, and the remaining residues non-transparent surfaces colored by atom charge (red, negative; blue positive, partial charges colored with hues in-between), in order to appreciate the positioning and orientation of the selected β-chain residues; same atomic color conventions as in (**A**) (except for carbon that is in green).

Our study measured six autoantibodies among newly diagnosed patients, i.e., IAA, GADA, IA-2A, ZnT8RA, ZnT8WA and ZnT8QA, which were scored as positive or negative. Focusing on 11 DQ motifs observed more than 10 times among patients, we evaluated the association of each DQ motif with the positivity for each type of autoantibody (Table 6). T1D subjects with the motif “DCAA-YSARD”, corresponding to DQ2.5, tended to have an increased frequency of GADA (OR = 1.56; p = 6.35*10^−8^) but a depressed frequency of IA-2A (OR = 0.59, p = 6.55*10^−11^). Among subjects with the motif “DQAA-YYARD” corresponding to DQ8.1, patients had a reversed association pattern, i.e., depressed GADA (OR = 0.87; p = 4.50*10^−3^) but elevated IA-2A (OR = 1.52, p = 2.18*10^−9^) frequency. Importantly, subjects with the motif “DQDA-YYARD”, corresponding to a slightly different DQ8.1 allele, had an elevated IA-2A positivity (OR = 5.67, p = 5.42*10–4), while its association with GADA was neutral (OR = 0.91, p = 0.67). Lastly, the motif “DRAA-YHVRD” corresponding to DQ6.4 (itself a neutral allele regarding T1D susceptibility) seemed to associate with increased frequency of ZnT8A (RA, WA and QA; OR = 1.92, 1.72 and 1.57, p = 6.25*10^−3^, 0.017 and 0.041, respectively), while having negative associations with GADA and IA-2A frequencies. In order to delineate possible pathways for T1D manifestation and resistance via islet-autoantigen-specific epitope presentation restricted by susceptible and resistant motifs, respectively, we have chosen three examples of: a susceptible (DQ8—InsB11-23), a neutral (DQB1*06:04—InsB5-15) and a resistant (DQB1*06:02—InsB5-15) heterodimer (Fig. 4A–C, and Supplementary Fig. S3A-C)^22,23^.

**Table 6:**
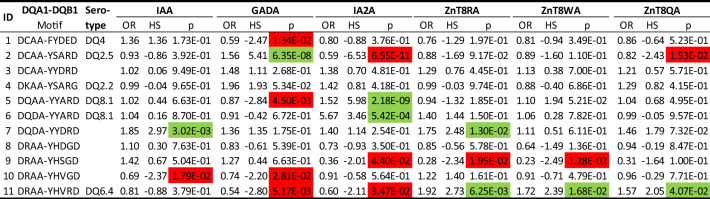
Association analysis of DQ motifs with elevated autoantibody levels among all patients (with five or more subjects): estimated odds ratio, haplotype score, and p-value, across all motifs with six autoantibodies (IAA, GADA, IA2A, ZnT8RA, ZnT8WA and ZnT8WA).

### Estimated attributable fractions of risk and protection from respectively susceptibility and resistance motifs

To quantify an overall T1D risk or protection from DQ motifs, we computed attributable risk and attributable resistance, respectively (methodological details of which are given in “Methods”). Briefly, we pooled DQ motifs into three groups: resistant, neutral or risk. Each individual had pairs of resistant, neutral or risk motifs. Using a logistic regression model on genotypes, we estimated regression coefficients corresponding to five genotypes, treating homozygous neutral motifs as the reference (Table 7). Motif frequencies were estimated from the controls (Table 7). In comparison with the reference genotype, in subjects with the homozygous resistant motif, the attributable resistance fraction was 25%, while its heterozygote with neutral motif had the resistance fraction of 7%. On the other hand, for subjects with a homozygous risk motif, the attributable risk fraction was 43%, and its heterozygote with the neutral motif had 19% attributable risk fraction. The heterozygote of resistant and risk motifs had 11% attributable risk fraction.

**Table 7:**
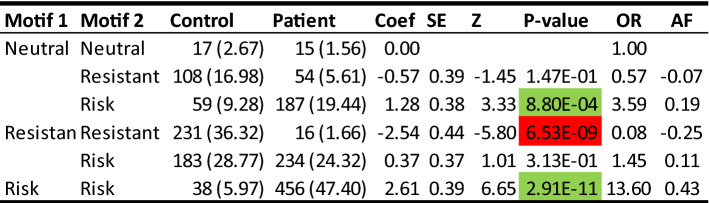
Estimated attributable fractions for all susceptible and resistant DQ motif genotypes: genotype frequency (%) among control and patient, estimated coef, standard error, Z-score, p-value, estimated odds ratio and attribuable fraction.

Notations and Conventions: As in Supplementary Fig. S1. Additional symbols are: For the putative homodimerization patch of β49-55 residues are marked with @ at the bottom of the sequence and highlighted in light green. Homodimerization of HLA-DQ has never been observed in any of the respective crystal structures. Yet, F(ab')2 fragment of anti-HLA-DQ antibodies differentially ligate HLA-DQ molecules on the surface of human monocytes and lead to differential (compared to HLA-DR and –DP) downstream signal transduction.

### Structural properties of nine DQ residues

Of the nine residues, least is known about the putative structural role of αa1Asp/Gly, the second residue of the mature HLA-DQ alpha chain. It is a residue without counterpart in HLA-DR alpha chains^21^. Of the remaining residues α44 is near pocket 1, while β9, β30, β57, and β70 are part of pockets 9, 6, 9 and 4 respectively, thus being among the main determinants for the binding of autoantigenic epitopes to the respective HLA-DQ molecules^20,22–24^. Further, β70 may also be considered as a most probable TCR contact residue and β57 as a potential such residue^23^. Residue β135 is part of the β134-148 peptide stretch shown to bind to co-receptor CD4 both in HLA-DR- as well as in mouse I-A-expressing APCs^24,25^. Along the same lines, α157 is a participant in the formation of the cognate TCR-induced pMHCII homodimer of heterodimers^26,27^, while α196 is within the membrane of the APC, contributes to signal transmission to such a cell, once engagement with cognate TCR is made^28^.

## Discussion

*HLA-DQ* genes are known to significantly contribute to susceptibility to islet autoimmunity and further to the progression to clinical onset of T1D, independently or jointly with *HLA-DRB1*. The major finding through exploration of the protein sequences of DQA1 and DQB1^12,13^ was that we identified, respectively, three and seven critical amino acid residues associated with T1D onset^14,15^. Integrating both sets of critical residues resulted in four alpha residues (αa1, α44, α157, α196) and five beta residues (β9, β30, β57, β70, β135) forming motifs that was associated with either susceptibility or resistance to T1D. The importance of both DQα and DQβ chains in determining T1D susceptibility and resistance is demonstrated not just in the respective motif consisting of 4 α-chain and 5 β-chain residues but also in all DQαβ combinations revealed by next generation targeted sequencing^11,12^. The deep sequencing approach reveals the importance of HLA-DQA-B combinations in T1D both susceptibility and resistance to considerations of –DQB or –DQA alone. This is evident, for example, in motif 5 (DQA1*02:01-B1*02:02), a T1D mildly resistant allele compared to the rare motif 9 (DQA1*03:02-B1*02:02) with only six copies among T1D patients, despite their sharing of an identical β-chain.

When interpreting association results of nine identified residues with respect to their binding properties, we should be mindful of two residue sets: one set includes all monomorphic and conserved residues, and another set includes those monomorphic residues in their respective clusters of DQ haplotypes. The former set of residues essentially defines the overall DQ molecule structure, and the latter set defines substructures of DQ heterodimers, e.g., DQ2.5 or DQ8.1 heterodimers. Hence, they remain structurally and functionally important, even though they may have limited associations with T1D risk.

Specifically, the newly identified DQB1*02:02, previously scored as DQB1*02:01 with low resolution methods, was found in a T1D-resistant combination (motif 5, Table 2, DQA1*02:01-B1*02:02) in 78 controls and 41 patients. The same β-chain by contrast, forms a rare haplotype in combination with a different α-chain, i.e. DQA1*03:02-B1*02:02, found only in six patients but in no controls. Similar striking findings were observed with DQB1*03:01 and DQB1*03:03. When either of them pairs with DQA1*03:01 they form rare haplotypes found only in 9 controls (motif 8, Table 2). Yet when the first of them pairs with DQA1*03:03 and the second with DQA1*03:02 they result in neutral haplotypes, found in 67 controls and 88 patients (motif 11, Table 2).

We also demonstrate that the DQ motifs associated with T1D appeared to be mediated through the frequency of islet autoantibodies. In particular, subjects with the DQ*DCAA-YSARD motif, corresponding to DQ2.5, more often had GADA (p = 6.35*10^−8^) than IA-2A (p = 6.55*10^−11^). Meanwhile, subjects with DQ*DQAA-YYARD and DQ*DQDA-YYARD, corresponding to DQ8.1, more often had IA-2A (p = 2.18*10^−9^ and 5.42*10^−4^, respectively), but less often GADA (p = 4.50*10^−3^). Other motifs had more variable associations with the different autoantibodies. To assess motif associations with the number of autoantibodies at diagnosis, we compared patients with 2, 3 or 4 autoantibodies with patients with 0 or 1 autoantibody, which revealed a single significant association (see Supplementary Table S4). In this neutral/mildly resistance motif, DQ*DQDA-YYDRD, the association with T1D was significant only when all four autoantibodies were present. These data suggest that the presence of all four autoantibodies overcame the resistance mechanism(s) of the DQ7 and DQ9 haplotypes (both β57Asp^+^) represented in this motif^29^. Hence, it cannot be excluded that other non-HLA genetic factors important to the pathogenesis contributed to autoantigen spreading and appearance of multiple autoantibodies.

It is important to realize that antigen-specificity in HLA-DQ- and -DR-directed autoimmune response (as well as resistance to it) is paramount for an organ-specific autoimmune disease, such as T1D. This is reflected by the observation that 4 of the 9 selected HLA-DQ residues are in anchoring pockets involved directly in antigen binding (β9, β30, β57, β70), another one (αa1) is near pocket 1, and two of the first four residues (β57, β70) in probable cognate TCR engagement and activation^23,30,31^. The remaining polymorphic residues (except αa1 about which there is hardly any information), are involved in other accessory functions of the HLA-DQ heterodimer: α135 in CD4 co-receptor binding, as part of the β134-148 stretch^32,33^, α157 in the formation of the putative homodimer of heterodimers that presumably facilitates CD4 binding^34,35^ and consequent cognate TCR activation and HLA-DQ signal transduction in the specific T cell and APC respectively^23^. Last, α196 is within the single membrane spanning domain of the respective chain and is involved in consequent signal transduction after the crucial step of pMHCII recognition by TCR, as just described^23,36^. The structural depictions of a susceptible, a resistant and a neutral HLA-DQ heterodimer happen to have bound peptide fragments from the same antigenic stretch of the insulin B-chain peptide 5–23^21,22,37,38^. Within the insulin peptide B5-23, the InsB11-23 epitope binds to DQ8 and is recognized by cognate CD4^+^ Th1 cells^37,38^ and the InsB5-15 epitope that binds in an identical register to the T1D-resistant heterodimer containing DQB1*06:02, as well as the neutral DQB1*06:04^22^. While not all epitopes have been compared in one-to-one comparisons between a resistant and a neutral/susceptible allele, it has already been shown that DQB1*06:02 can out-compete DQ8 for the epitope InsB5-23 by forming the DQB1*06:02—InsB6-14 complex, with the peptide shown only as the core nonamer^22^. The same is probably the case for the neutral allele DQB1*06:04, which binds the same preproinsulin epitopes as DQB1*06:02, albeit at slightly higher IC50 values, i.e. with lower affinity^22^. It will naturally be of interest to determine how newly-identified risk and resistant alleles in the Swedish population interact with T1D autoantigenic epitopes^12,13^.

Two recent studies have added significant knowledge to the area of HLA-DR-DQ-restricted proinsulin and GAD65 epitopes^26,27^. The first study deals with proinsulin-specific epitopes and their cognate CD4 + T cell clones found in healthy children homozygous for either HLA-DR3-DQ2, DR4-DQ8 or being DR3-DQ2/DR4-DQ8 heterozygotes. It is based on the observation that IAA represents the first appearing autoantibody in about 50% of children with these high-risk HLA-DR-DQ haplotypes who later on developed additional islet autoantibodies followed later by clinical onset of T1D^39,40^. Remarkably, the epitopes were identified using PBMCs from these healthy children and relevant antigen-presenting cells supplemented with various proinsulin antigenic peptides. IL-2 combined with IL-7 was used to stimulate both CD4^+^ Teffectors as well as CD4^+^ T regulatory cells (Tregs). The second study deals with the effects of GAD65-alum treated newly-diagnosed T1D patients by stimulating frozen and thawed PBMCs in culture with whole GAD65 and antigen-specific bifunctional Th1/Th2 cell lines generated subsequently^27^. It is noteworthy that the T cell lines generated from heterozygous HLA-DR3-DQ2/DR4-DQ8 individuals were greatly restricted in the number of recognized epitopes, distinctly fewer than the sum of those recognized by T cell lines restricted to either HLA-DR3-DQ2 or HLA-DR4-DQ8^27^. The specific HLA class II restricting element(s) of the respective CD4 + T cell lines and clones were not determined. Interestingly, a recent study showed that GAD65- and IGRP (islet glucose-reactive protein)-specific CD4^+^ T effector and T regulatory cells (Tregs) were present at higher frequencies in the peripheral blood of HLA-DRB1*15:01-DQA1*01:02-B1*06:02 positive individuals (i.e. negatively associated with T1D) compared to subjects with neutral or T1D-susceptible alleles^28^. Furthermore, cloned Tregs carrying these specificities and restricted to HLA-DRB1*15:01 suppressed so-restricted and specific Teffectors. It will be of interest to determine whether HLA-DQ alleles linked with resistance to T1D can also give rise to similar Tregs. A similar effect has already been noted in the case of Goodpasture’s syndrome with higher frequency of Tregs specific for the Collagen IV autoantigen α3_135-145_ in HLA-DRB1*01:01^+^ (disease-resistant) individuals, compared to HLA-DRB1*15:01^+^ (disease-susceptible) individuals, regarding the same autoantigen specificity, albeit with binding register shifted by one residue^41^. This would extend the “epitope-stealing” effect, long hypothesized for T1D and already documented for HLA-DQA1*01:02-B1*06:02 over HLA-DQ8-specific proinsulin epitopes^25,37,42^. If DQA1*01:02-B1*06:02-restricted Tregs can be shown to have similar suppressive effects as those restricted to DRB1*15:01 above, then the contributions of the former molecule to T1D resistance would be multiple. The amino acid motifs identified in the present study should prove useful to identify these mechanisms.

All our considerations thus far, have assumed equal levels of membrane HLA-DQ protein expression on APCs. It was shown over two decades ago, that the density of APC, the level of expression of HLA-DQ on the membrane of such APC, as well as the level of soluble peptide antigen present, would influence the proliferation of restricted and specific CD4 + T effector cells^43^. While the upstream regions of HLA-DQA and –DQB genes are replete with regulatory nucleotide sequences, very few studies exist on the regulation of HLA-DQ membrane protein expression by the various different alleles^44–46^. Notwithstanding the fact that in a disease situation (i.e. the islet β cells under autoimmune attack, as well as the proximal pancreatic lymph nodes) the cytokine and chemokine milieu is an unknown and may differentially influence the expression of HLA-DR-DQ heterodimers even in an allele-specific manner. The situation has become more complex by the fact that human, as well as mouse, islet β cells, are closely approximated by islet macrophages that receive antigenic peptide fragments from these β cells and possess MHCII molecules through which such antigens are presented^47,48^. It is also of interest that Tregs, shown to be suppressive over Teffector cells specific for T1D autoantigens, seem to be consistently absent from islets in autopsy material from newly diagnosed T1D patients^49,50^.

We have limited our discussion to possible autoantigenic epitopes and their interactions with HLA-DQ molecules and alleles that confer susceptibility or resistance to, or are neutral to T1D. Unfortunately nothing can be said about the interaction of diabetogenic pHLA-DQ heterodimers with cognate TCRs, as there is no single complex with solved 3-dimensional coordinates. Such complex would provide information about the possible engagement of the amino acid residue motifs identified in the present study. Using TCR—HLA-DR-DQ complexes with CNS antigenic epitopes in multiple sclerosis as a guide (the only available such complexes from organ-specific autoimmune diseases), the TCR orientation with respect to pMHCII is not the canonical diagonal, but expected to be off-diagonal^24^. The fact that this interaction, that leads to autoimmunity, is different from the canonical one is also reflected in the irregular form of immunological synapse formed by autoimmune CD4^+^ T cell clones from patients with multiple sclerosis and T1D, compared to such clones specific for influenza virus peptides: there was no accumulation of pMHCII complexes in the central Supramolecular Activation Cluster of the synapse; nor for that matter, any transport of TCR-pMHCII complexes into this central cluster of the synapse^51^. In another example, the key HLA class II molecule accounting for over 90% of patients with celiac disease is DQ5.2 (A1*05:01-B1*02:01), while the very closely related DQ2.2 (A1*02:01-B1*02:02) is neutral. The α22Y/F dimorphism (DQ5.2 compared to DQ2.2, respectively), differentiate between the binding at p3 of gluten peptides in DQ2.2 compared to by DQ2.5. The latter combinations select for a very focused set of cognate TCRs that are also pathogenic for self resulting in small intestinal tissue destruction^52^. The small set of gluten peptide-DQ2.2 complexes surprisingly resulted in a wide set of cognate TCRs, none of which were pathogenic^52^.

A potential weakness to the present studies is that all children are Swedish, only 8% in the present cohort were born to parents born in another country along with the grandparents^29^ and that the environmental exposure would be relatively homogeneous. It cannot be excluded that the pattern of amino acid residues observed as well as the presence of autoantibodies at the time of clinical onset may reflect a Swedish environment. The exposures of these children diagnosed with T1D are not only long term but also multiplex as the incidence rate for IAA-first in DR4-DQ8 children showed a peak at 1–3 years of age in the TEDDY study as related to prolonged shedding of enterovirus^40,53,54^ and other studies have shown that viral exposure of islet autoantibody positive children may accelerate progression to clinical onset^55^. It cannot be excluded that the two potential endotypes in T1D etiology and multiple environmental exposures leading to the eventual clinical onset of T1D have been important to the frequency of specific amino acid motifs.

Three additional limitations of the study are worth noting. The current study uses the retrospective case–control approach, drawing patients from clinics around Sweden and geographically matched controls. Inevitably, hospital-referred patterns may bias patient selection, and some controls, if any, may have not developed T1D at the time of the study. However, both issues are unlikely to alter the results of assessing the genetic susceptibility to T1D, because HLA genes are time-invariant, T1D is relatively uncommon among adults and healthcare system provides universal care in Sweden. Another limitation is associated with measuring autoantibodies among patients at the time of diagnosis. As noted above, autoantibodies are important biomarkers predicting T1D onset^54,56^ but have variable temporal patterns. In the absence of observing such termporalities, the current investigation of genetic associations with autoantibodies is inevitably limited and needs to be cautiously interpreted, even though observed associations still remain meaningful. The third limitation concerns the possibility of genetic heterogeneity due to immigrants that may confound the association results. In the BDD study, 8% of patients are children born to immigrants^16,29^. Even though immigrant status information is not controlled, it is expected that normal controls may have a comparable percentage of immigrants. Given the relatively small percentage, the mixture of immigrants with native Sweden is not expected to substantially alter association results observed here.

Recently, a novel reductionist conditional approach was used to search for critical residues^57^. The key idea is to repeat residue-specific association tests, while adjusting (conditioning on) selected residues^57^. Their conditional analysis led to identification of β57 as a critical residue for T1D. However, this approach fails to differentiate between empirically observed associations that result from genetic causal associations or from LD-induced associations. Hence their results have invited much controversy. In contrast to the reductionist approach, our approach, being a holistic, starts with motifs of all polymorphic residues, retaining their sequence structure, and eliminates those residues that do not associate with T1D within their respective clusters. Consequently, our primary results concern T1D associations with motifs of selected residues, and these motifs explain nearly all DQ haplotype associations with T1D. By the same reason, this holistic approach is probably preferred to other residue-specific association methods.

In T1D, recent data in the TEDDY study^53^ suggest that prolonged shedding of enterovirus B in young DR4-DQ8.1 positive children preced the appearance of insulin autoantibodies (IAA) as a biomarker for autoimmunity against the beta cells. In older children, prolonged shedding of adenovirus F in DR3-DQ2.5 positive children preceded the appearance of GAD65 autoantibodies (GADA) as a first biomarker for beta cell autoimmunity. The intersection between prolonged virus exposure and appearance of a first autoantibody marker is yet to be dissected. However, it cannot be excluded that the motifs described in the present paper contribute to antigen presentation that involves a competition between a viral peptide or either (pro)insulin or GAD65 to trigger an autoimmune rather than a viral immune response. It is therefore of interest that the susceptibility motifs were more diverse among controls than among patients (entropy = 2.5 versus 1.85, respectively). Out of 25 unique motifs, three motifs “DCAA-YSARD”, “DQAA-YYARD”, and “DQDA-YYARD” exhibited significant susceptibility to T1D (OR = 2.10, 3.34 and 3.71, p = 1.96*10^−20^, 2.69*10^−72^ and 1.53*10^−6^, respectively) with one uncommon motif “DQDA-YSARG” was observed seven times only among patients. On the other hand, eight motifs “DCAA-YYDRD”, “DKAA-YSARG”, “DKAA-YYDRD”, “DRAA-FYDGD”, “DRAA-LYDRD”, “DRAA-YHDGD”, “DRAA-YHVGD” and “GRAT-YHDGD” were negatively associated with autoimmunity against β cells resulting in T1D (OR = 0.19, 0.35, 0.09, 0.03, 0.07, 0.25, 0.80 and 0.08, p = 2.32*10^−25^, 8.47*10^−9^, 1.99*10^−10^, 2.25*10^−60^, 1.14*10^−3^, 7.72*10^−19^, 0.0312 and 6.38*10^−3^, respectively), with two motifs “DCSA-YYDRD” and “DQAA-YYDRD” respectively observed 7 and 9 times among only controls. It is obvious that the lower HLA-DQ motif entropy among T1D patients reflects the fact that susceptibility to T1D requires certain structural features on the part of the susceptibility-conferring HLA-DQ molecules. These certainly would have to do with the relevant autoantigenic epitopes, as well as the molecules that the T1D-causing pMHCII complexes interact with such cognate TCRs and CD4 co-stimulatory molecules, and perhaps other molecules in the respective signal transducing pathways.

## Methods

### Study design and populations

We used a case–control study design to evaluate genetic associations with T1D. A total of 962 patients (cases) were from the nation-wide Swedish Better Diabetes Diagnosis (BDD) study^16,58,59^, which involves participation in 2005–2010 of all 42 pediatric clinics in Sweden. The American Diabetes Association and World Health Organization criteria were used for the diagnosis of diabetes and to classify the disease^60^. Included patients had one or several autoantibodies against either insulin (IAA), GAD65 (GADA), IA-2 (IA-2A), and three variants (amino acid 325 being either R, W or Q, ZnT8-RA, ZnT8-WA or ZnT8-QA, respectively) at the time of clinical diagnosis^16,58^. Patients diagnosed with diabetes at 9 months–18 years of age were sequentially enrolled in the BDD study^16,29,61^. A total of 636 nation-wide and geographically representative controls were analyzed at the same time^62^. The demographic characteristics of the BDD patients are detailed elsewhere^16,29,59^. Approximately 8% of BDD participants are immigrants^16,29^. The informed consent was taken from participants/guardians for young participants (< 18 years old). The Karolinska Institute Ethics Board approved the BDD study (2004/1:9).

All experiments (DNA extraction, genotyping, measuring islet autoantibodies) and analytic methods are carried out in clinical laboratories (CLIA certified or equivalent in Sweden), following relevant guidelines and regulations.

### DNA extraction

The plasmid Max isolation kit (Qiagen, Bothell, Washington, USA) was used to isolate DNA according to the manufacturer’s instructions from frozen whole blood samples of cases and controls.

### HLA next generation targeted sequencing (NGTS) analysis

The NGTS HLA typing approach utilized PCR based amplification of HLA and sequencing using Illumina MiSeq technology as described in detail^63,64^. Briefly, the laboratory steps consisted of consecutive amplicon-based PCR with bar coding incorporated in the PCRs for individual sample tracking followed by application to the MiSeq. Robust assays for each of the target loci for all class II genes were purchased from Scisco Genetics Inc., Seattle WA (https://sciscogenetics.com). This system employs amplicons individually extending 4–500 bp segments covering each of exons 1 ~ 4 of HLA-DQA1 and -DQB1 and MiSeq read depths of over 100-fold coverage for each amplicon were obtained. The analytical tools to define haplotypes and genotypes were made available as part of the genotyping kit assay from Scisco Genetics Inc. (Seattle, WA). To date these tools have been tested—with 100% accuracy—on > 2000 control samples genotyped with the present NGS approach^63,64^.

### Amino acid sequences of DQA1 and DQB1 alleles

As NGTS sequenced DNA nucleotides of selected exons and used sequences to infer DQA1 and DQB1 alleles, we determined HLA-DQA1 and -DQB1 genotypes at the high resolution of 6 digits or higher, based on the HLA nomenclature of IMGT (https://raw.githubusercontent.com/ANHIG/IMGTHLA/Latest/alignments/DQA1). Further, we determined physical positions of individual aminoacids in the alpha chain from codon α1 to α232, and in the beta chain from codon β1 to β237. We have adopted the numbering system as first suggested^65^. and later improved^20^, that essentially allows for structural equivalence among residues of various MHC II alleles, regardless of gene locus or species, and based on the structure of the first published MHC II allele HLA-DR1^34,66^.

### Islet autoantibodies

GADA, IA-2A, IAA, and three variants of ZnT8A (ZnT8-RA, ZnT8-WA or ZnT8-QA, respectively) were determined in quantitative radio-binding assays using in house standards to determine levels as previously described in detail^16,58^.

### Cis- and trans-heterodimer through interlocus recombination

High LD enables us to haplotype HLA-DQA1 and DQB1 genotypes with exceptionally high posterior probabilities (minimum value > 0.97). Let $(\dot{h}/\ddot{h}) = (\dot{a}_{A} \dot{a}_{B} /\ddot{a}_{A} \ddot{a}_{B} )$ denote a pair of -DQA1 and -DQB1 haplotypes, in which $\dot{a}_{A} \dot{a}_{B}$ and $\ddot{a}_{A} \ddot{a}_{B}$ are two cis-haplotypes (or cis-heterodimers). In contrast, two trans-haplotypes are $\dot{a}_{A} \ddot{a}_{B}$ and $\dot{a}_{A} \ddot{a}_{B}$. In case that -DQA1 and -DQB1 are doubly homozygous, all four haplotypes are identical. For a semi-heterozygous, two trans-haplotypes are identical to two cis-haplotypes. Thirdly, a doubly heterozygous DQA1 and DQB1 would lead to four different haplotypes. Under the assumption that the ”interlocus recombination” is a completely random process, alleles of DQA1 and DQB1 are always recombined to form cis- and trans-heterodimers within cells, even though some trans-heterodimers may be structurally prohibited or unexpressed^18^. Lastly, it is expected that some trans-heterodimers, from doubly heterozygotes, are novel and unobserved in the general population, while many others could be observed in the general population. There are still some that were probably expressed at the mRNA level but were not permitted to form proteins^18^.

### Conditional analysis strategy

In the empirical association analysis of T1D disease outcome ($Y$) with one primary covariable $X$ in light of another correlated variable $Z$ that may potentially confound the association of interest, the conventional approach is to adjust the confounding variable in the association analysis via the following logistic regression model for modeling the disease probability:

$$\Pr (Y = 1|X,Z) = 1/\{ 1 + \exp [ - {\text{adjust}}(X,\beta ) - {\text{test}}(Z,\gamma )]\} ,$$

in which the first component “${\text{adjust}}(X,\beta )$” is a function of the primary covariates to be adjusted for and is indexed by the parameter $\beta$, and the second component ”${\text{test}}(Z,\gamma )$” is a function of the test covariates indexed by the parameter $\gamma$. Here the first step is to fit a logistic regression model with the “${\text{adjust}}(X,\beta )$”. Then fixing this component, the second step is to systematically test the disease association with the second component ${\text{test}}(Z,\gamma )$. Despite the intuitive appeal of the conditional analysis for adjusting the primary variable, it also has several intrinsic limitations, since this adjustment is empirical rather than not biological (see “Discussion”). Nevertheless, it is helpful to perform such a conditional analysis for the following scenarios:

1. Assessing disease associations with trans-motifs, after adjusting for the cis-motifs present.
2. Assessing disease associations with individual residues, after adjusting for cis-motifs

### Shannon’s entropy

HLA-DQ motifs are polymorphic, and have a degree of diversity. Because of their categorical nature, we use the information entropy, a basic information unit in the information theory, which is also known as Shannon’s entropy introduced by Claude Shannon^21^. Let $f_{1} ,f_{2} , \ldots ,f_{q}$ denote allelic frequencies for *q* alleles. The corresponding Shannon’s entropy is defined as

$$Entropy = - \sum\limits_{j = 1}^{q} {} f_{j} \log (f_{j} ).$$

### Attributable risk/protection fractions

Attributable fraction is used to quantify an overall fraction of risk (protection) that can be accounted by a binary exposure factor, i.e., exposed versus non-exposed, and depends on the magnitude of the association and frequency of the exposure in the population^67^ which can be computed as:

$$AF_{jk} = \frac{{f_{jk} (OR_{jk} - 1)}}{{1 + f_{jk} |OR_{jk} - 1|}},$$

which may be referred to as attributable risk fraction (AF > 0) or attributable protective fraction (AF < 0).

### Structural properties of HLA-DQ residues

All depictions of determined structures of various HLA-DQ alleles have been carried out using the WebLabViewer v. 3.5 and the DSViewerPro v. 6.0, 3-D molecular rendering software of Accelrys (currently Dassault Systèmes, BIOVIA, San Diego, CA, USA, https://www.3ds.com/products-services/biovia), based on coordinates freely available in the Protein Data Bank. The DQ8 (A1*03:01-B1*03:02)—insulin B11-23 complex structure is obtained from the determined crystal structure^22^. The modeled structures of the complexes of DQB1*06:02 (A1*01:02-B1*06:02)—insulin B4-16, and DQB1*06:04 (A1*01:02-B1*06:04)—insulin B4-16 are obtained from the crystal structure of DQB602-hypocretin complex, as already described^23^.

### Statistical analysis and software

Upon extracting aminoacids for each DQ haplotype, we compute distances between all unique sequences pairwise by the stringdist function of R package, with the Levenshtein distance, and then apply the hierarchical clustering algorithm using the hclust function of R package (version 4.0.2, https://www.r-project.org) with the agglomeration method of ward.D2, i.e., clustering two sequences closer together, if their sum of squared distances is relativly small. For the association analysis, we use the logistic regression function in “glm” of R package. To assess their associations with qualitatively determined elevations of islet autoantibodies (IAA, GADA, IA-2A, ZnT8RA, ZnT8WA, and ZnT8QA), we also use haplo.cc. To address the instability of “small sample sizes” in the diplotypic association analysis, we compare frequency counts of one diplotype versus those of all other diplotypes combined, and compute the corresponding odds ratios, 95% confidence interval, and the Fisher’s exact test p-values.

With respect to p-values and multiple comparison issues, we took an unconventional approach, presenting the p-values without the multiple comparison correction. While the conventional approach was to compute corrected p-values for number of comparisons by, say the Bonferroni correction or false discovery rate^68^, its intension was to control the overall false positive error rates of all comparisons. In the current context with the analysis of HLA-DQ haplotypes/diplotypes, the roles of both genes in T1D were implicated in multiple empirical and functional studies. Further, the empirical haplotype association analysis suggested that DQ associations are highly significant at the 5% level, even if multiple comparisons with 45 comparisons are considered. Hence, the haplotypic and diplotypic association analyses aimed to uncover which haplotypes/diplotypes may explain the overall association, i.e., our explorations were in the alternative hypothesis domain. Finally, p-values with no multiple comparison corrections have clear and simple interpretations, free from varying haplotypes/diplotypes in various explorations. Throughout the analyses, we used the threshold of 0.05 to highlight those p-values to be positively or negatively associated, while being mindful that some p-values, close to 0.05, could be falsely labeled. Naïve applications of any multiple comparisons without varying correction factors could obscure the interpretations of results.

When computing haplotype-specific odds ratios, we intentionally avoided choosing a specific reference haplotype, because any choice of a particular reference haplotype could have undesired implications in the interpretations of the results. Instead, we considered two different strategies. One way was to compute the odds ratio of one haplotype versus all other haplotypes. Alternatively, we choose a “virtual haplotype” that has no association with T1D. As expected, the ratio of its corresponding haplotype frequency among patients over that among controls equals one. Hence, the expected odds ratio for any given haplotype is thus computed as the ratio of two observed haplotype frequencies among patients and controls^14^.

## Supplementary Information

Supplementary Information 1.

Supplementary Information 2.

Supplementary Information 3.

Supplementary Information 4.

Supplementary Information 5.

Supplementary Information 6.

Supplementary Information 7.
