# Supplementary material for: Nine residues in HLA-DQ molecules determine with susceptibility and resistance to type 1 diabetes among young children in Sweden
Source: Sci Rep. 2021 Apr 23;11:8821. doi: 10.1038/s41598-021-86229-8 (PMC8065060; doi:10.1038/s41598-021-86229-8)
Supplement: Supplementary file 1 — Supplementary Information 1. [file 41598_2021_86229_MOESM1_ESM.docx]

**Nine residues in HLA-DQ molecules determine with susceptibility and resistance to type 1 diabetes among young children in Sweden**

Lue Ping Zhao,^1+^ George K Papadopoulos,^2*^ Antonis K. Moustakas,^3^ George P. Bondinas,^2**^ Annelie Carlsson,^4^ Helena Elding Larsson,^5^ Johnny Ludvigsson,^6^ Claude Marcus,^7^ Martina Persson ^8^, Ulf Samuelsson,^6^ Ruihan Wang,^9^ Chul-Woo Pyo,^9^ Daniel E. Geraghty,^9^ and Åke Lernmark^5*^

1. Public Health Sciences Division, Fred Hutchinson Cancer Research Center, Seattle, WA, USA
2. Laboratory of Biophysics, Biochemistry, Biomaterials and Bioprocessing, Faculty of Agricultural Technology, Technological Educational Institute of Epirus, GR47100 Arta, GREECE
3. Department of Food Science and Technology, Faculty of Environmental Sciences, Ionian University, GR26100 Argostoli, Cephalonia, Greece
4. Department of Pediatrics, Lund University, Lund, Sweden.
5. Department of Clinical Sciences, Lund University CRC, Skåne University Hospital, Malmö, Sweden.
6. Crown Princess Victoria Children´s Hospital, Region Östergötland and Div of Pediatrics, Department of Clinical and Experimental Medicine, Linköping University, Linköping, Sweden.
7. Department of Clinical Science and Education Karolinska Institutet and Institution of Medicine, Clinical Epidemiology, Karolinska Institutet, Stockholm, Sweden
8. Department of Medicine, Clinical Epidemiological Unit, Karolinska Institutet, Stockholm, Sweden
9. Clinical Research Division, Fred Hutchinson Cancer Research Center, Seattle, WA, USA.

^+^Corresponding author, [lzhao@fredhutch.org](mailto:lzhao@fredhutch.org)

* GKP retired from the Technological Educational Institute (TEI) of Epirus, GR47100 Arta, GREECE on September 1^st^, 2018. The address is given for identification purposes only. As of October 1^st^, 2018, the TEI of Epirus has been absorbed by the University of Ioannina. The respective department is now called Department of Agriculture.

** Adjunct member of the laboratory

**SUPPLEMENTARY FIGURE LEGENDS**

Supplementary Figure S1. Amino acid sequences of HLA-DQA1* alleles recorded in this study, alongside established functional associations for given residues or groups thereof.

Supplementary Figure S2. Amino acid sequences of HLA-DQB1* alleles recorded in this study, alongside established functional associations for given residues or groups thereof.

Supplementary Figure S3. Detailed TCR views of pockets 4, 6, 7 and 9 from each of Fig. 4A-C. A. DQ8-InsB11-23, B. DQA1*01:02-B1*06:04—InsB4-16, C. DQA1*01:02-B1*06:02—InsB4-16. All residues are in stick form of the same thickness, with the same color and depiction conventions as in Fig. 4B, C. Each HLA-DQ residue shaping one or more pockets influences, subtly or radically, the anchor residue preference for the antigenic peptide. For example, in the closely related molecules DQA1*01:02-B1*06:04 (T1D neutral, Figure S4B) and DQA1*01:02-B1*06:02 (T1D resistant, Figure S4C), β30His in the former allows for additional acidic Asp anchor but forbids Pro at p6 that are respectively forbidden/allowed in the latter with β30Tyr, while both may participate in hydrogen bonding with the antigenic peptide backbone; β57Val in the former DQ molecule allows for aliphatic, Tyr/Phe and less so acidic anchors in pocket 9 , while β57Asp in the latter one permits only small aliphatic and less so acidic residues ^23^. The β57Asp residue, in contrast to non-Asp substitutions greatly influences the stability of pMHCII complexes by forming a salt bridge with α76Arg and at least one hydrogen bond with the antigenic peptide backbone^22,27,28,33^. The majority of β57non-Asp HLA-DQ molecules have Ala/Ser in this position, that promotes the binding of an acidic anchor forming a salt bridge with α76Arg, as in Figure S3A ^22^. The other major T1D susceptible molecule, DQ2, that is not detailed here, besides β57Ala, has β28 Ser /β30Ser and β70Lys/β71Arg. The first three allow for wide preference (even acidic residues) at p6, and aromatic (and less so acidic) anchors at p9, while the last two allow for polar/acidic anchors at p4/p7 and forbid basic residues at these positions^53^.
