# Supplementary material for: Nine residues in HLA-DQ molecules determine with susceptibility and resistance to type 1 diabetes among young children in Sweden
Source: Sci Rep. 2021 Apr 23;11:8821. doi: 10.1038/s41598-021-86229-8 (PMC8065060; doi:10.1038/s41598-021-86229-8)
Supplement: Supplementary file 3 — Supplementary Information 3. [file 41598_2021_86229_MOESM3_ESM.docx]

**Supplementary Figure 1**

**§¶¦1 10 20 30 40 50 60 70 80 90**

**DRA*01:01 IKEE__IIQ-AEF_LNPDQ__EFMFD_____I_H__MAK___V__LE__GR_AS_EA____A_I__D_A__E__T__S_Y_PI__VP____**

DQA1*01:01:01 EDIVADHVASCGVNLYQFYGPSGQYTHEFDGDEEFYVDLERKETAWRWPEFSKFGGFDPQGALRNMAVAKHNLNIMIKRYNSTAATNEVPEVT

**DQA1*01:02:01 _________________________________Q___________________________________________________________**

DQA1*01:03 ________________________F________Q______K____________________________________________________

DQA1*01:04:01 _G___________________________________________________________________________________________

DQA1*01:07 _G____________________________________________________________________________C______________

DQA1*01:10 ________________________F________Q______K__________________________________________T_________

**DQA1*02:01 __________Y______S______F___________________V_KL_L_HRL-R____F__T_I__L______L___S_____________**

**DQA1*03:01:01** **__________Y______S_______S__________________V_QL_L_RR_RR____F__T_I__L______V___S_____________**

DQA1*03:02 __________Y______S_______S__________________V_QL_L_RR_RR____F__T_I__L______V___S_____________

DQA1*03:03 __________Y______S_______S__________________V_QL_L_RR_RR____F__T_I__L______V___S_____________

DQA1*04:01:01 __________Y______S_______________Q_____G____V_CL_VLRQ_-R____F__T_I__T______L___S_____________

DQA1*04:02 __________Y______S_______________Q_____G____V_CL_VLRQ_-R____F__T_I__T______L___S_____________

DQA1*04:04 *****_____Y______S_______________Q_____G____V_CL_VLRQ_-R____F__T_I__T______L___S_____________

**DQA1*05:01:01 __________Y______S_______________Q_____G____V_CL_VLRQ_-R____F__T_I__L_____SL___S_____________**

DQA1*05:03 __________Y______S_______________Q_____G____V_CL_VLRQ_-R____F__T_I__L_____SL___S_____________

DQA1*05:04 *****_____Y______S__L____________Q_____G____V_CL_VLRQ_-R____F__T_I__L_____SL___S_____________

DQA1*05:05 __________Y______S_______________Q_____G____V_CL_VLRQ_-R____F__T_I__L_____SL___S_______******

DQA1*05:09 K_________Y______S_______________Q_____G____V_CL_VLRQ_-R____F__T_I__L_____SL___S_____________

DQA1*05:10 *****_____Y______S_______________Q_____G____V_CL_VLRQ_-R____F__T_I__L_____SLV__S_____________

DQA1*060101 __________Y______S______F________Q_____G____V_CL_VLRQ_-R____F__T_I__T______L___S_____________

**~ 1 6 1 ~ 11 1 11 6 66 96 99 9 #**

**+ ! - ! !9 !**

**|______________________| ! ~**

**+**

**91 100 110 120 130 140 150 160 170 180**

**DRA*01:01** _LTN___E_RE__V___FI_KFT_________R__KP__T_____V__PRE__L_R_FH__P____TEDV___R_______E_______F

DQA1*01:01:01 VFSKSPVTLGQPNTLI**C**LVDNIFPPVVNITWLSNGQSVTEGVSETSFLSKSDHSFFKISYLTFLPSADEIYD**C**KVEHWGLDQPLLKHWEP

**DQA1*01:02:01 __________________________________________________________________________________________**

DQA1*01:03 ________________**_**__________________HA___________________________________**_**_________________

DQA1*01:04:01 ________________**_**_______________________________________________________**_**_________________

DQA1*01:07 ________________**_**_______________________________________________________**_**_________________

DQA1*01:10 ________________**_**__________________HA___________________________________**_**_________________

**DQA1*02:01 ___________________________________H_____________________________________________E________**

**DQA1*03:01:01** **___________________________________H_____________________________________________E________**

DQA1*03:02 ________________**_**__________________H______________________________D_____**_**________E________

DQA1*03:03 ________________**_**__________________H______________________________D_____**_**________E________

DQA1*04:01:01 ________________**_**__________________H____________________________________**_**________E________

DQA1*04:01:02 ________________**_**__________________H____________________________________**_**________E______**

DQA1*04:02 ________________**_**__________________H________I___________________________**_**________E______**

DQA1*04:04 ________________**_**__________________H_______________________H____________**_**________E______**

**DQA1*05:01:01 _____________I_____________________H__________________________L____E_S___________K________**

DQA1*05:03 _____________I__**_**__________________H__________________________L___SE_S___________K________

DQA1*05:04 ******************************************************************************************

DQA1*0505 _____________I_____________________H__________________________L____E_S__**_**________K________

DQA1*05:09 _____________I__**_**__________________H__________________________L____E_S__**_**________K________

DQA1*05:10 _____________I__**_**__________________H__________________________L____E_S__**_**________K______**

DQA1*06:01:01 ________________**_**__________________H____________________________________**_**________E________

**| # / / / / / / / ## #|/ / / /// # # #**

**| |**

**|__________________________S-S__________________________|**

**181 190 200 210 220 230**

**DRA*01:01** DA_S_LP_T__N_______T______II__I___K_V_KSN_AERR___

DQA1*01:01:01 EIPAPMSELTETVVCALGLSVGLVGIVVGTVFIIQGLRSVGASRHQGPL

**DQA1*01:02:01 _______________________M_________________________**

DQA1*01:03 _________________________________________________

DQA1*01:04:01 _______________T_________________________________

DQA1*01:07 _______________T_________________________________

DQA1*01:10 _________________________________________________

**DQA1*02:01 _______________________________L__R______________**

**DQA1*03:01:01 ___T___________________________L__R______________**

DQA1*03:02 ___T___________________________L__R______________

DQA1*03:03 ___T___________________________L__R______________

DQA1*04:01:01 __________________________________R______________

DQA1*04:02 *************************************************

DQA1*04:04 *************************************************

**DQA1*05:01:01** __________________________________R______________

DQA1*05:03 __________________________________R______________

DQA1*05:04 *************************************************

DQA1*05:05 __________________________________R______________

DQA1*05:09 __________________________________R______________

DQA1*05:10 *************************************************

DQA1*06:01:01 __________________________________R______________

**# 🡨--trans-membrane---🡪**

Notations and conventions:

1. Alleles with known crystal structure are in bold. These structures concern only the extracellular α1β1/α2β2 domains, bound to an antigenic peptide, and occasionally in complex with a cognate T cell receptor.

2. Symbols in the numbering:

**§**: αc1, **¶**: αb1, **¦**: αa1

3. Identity in residues is indicated by _, while unknown residues are shown as *, and deletions are marked as -.

4. Symbols below the sequence of the last allele:

For antigen binding (highlighted in yellow): residues participating in the formation of a particular pocket indicated by the number of the pocket (1, 4, 6, 7, 9) at the bottom of the column; in case a residue participates in more than one pockets this is indicated by numbers on two or three lines, accordingly. In molecules with a deletion in α52, residue α51 is partially reoriented so that it can participate in the formation of pocket 1. For interchain interactions: ! : residues forming hydrogen bonds with antigenic peptide backbone (highlighted in red); ~: residues forming an intra- or interchain salt bridge, marked with respective electric charge signs and highlighted in blue; # : residues participating in the formation of the putative homodimer of heterodimers (highlighted in pink); / : residues involved in the binding of CD4 (highlighted in turquoise). Disulfide bridges in orange. The intramembranous sequence is noted and highlighted in gray.

In order to help the reader keep track of which HLA-DQA proteins are allowed to structurally pair with given HLA-DQB proteins, compatible combinations are colored accordingly, either in purple (DQA1*01-DQB1*05/06) or in green (DQA1*02/03/04/05/06-DQB1*02/03/04).
