# Supplementary material for: Nine residues in HLA-DQ molecules determine with susceptibility and resistance to type 1 diabetes among young children in Sweden
Source: Sci Rep. 2021 Apr 23;11:8821. doi: 10.1038/s41598-021-86229-8 (PMC8065060; doi:10.1038/s41598-021-86229-8)
Supplement: Supplementary file 4 — Supplementary Information 4. [file 41598_2021_86229_MOESM4_ESM.docx]

**Supplementary Figure 2**

**1** **10 20 30 40 50 60 70 80 90**

**DRB1*01:01 G_TRPR_LW_L_FE_H_F_______LLE_C___Q__S________E_____EL___D________DL__QR__A__TY_____G_GESFT**

DQB1*05:01:01 **RDSPEDFVYQFKGLCYFTNGTERVRGVTRHIYNREEYVRFDSDVGVYRAVTPQGRPVAEYWNSQKEVLEGARASVDRVCRHNYEVAYRGI**

**DQB1*05:02:01 ________________________________________________________S_________________________________**

DQB1*05:03:01 ______________**_**_________________________________________D_____________________**_**___________

DQB1*05:04 ______________**_**______________Y__________________________S________DI__ED_______**_**___________

**DQB1*02:01:01 _____________M___________L_S_S______I________EF____LL_L_A________DI__RK__A_________QLEL_TT**

**DQB1*02:02 _____________M___________L_S_S______I________EF____LL_L_A________DI__RK__A_________QLEL_TT**

DQB1*03:01:01 ____________AM**_**__________Y___Y_______A______E_______L_P_D____________RT__EL_T_**_**____QLEL_TT

**DQB1*03:02:01 _____________M___________L___Y_______A______________L_P_A____________RT__EL_T______QLEL_TT**

DQB1*03:03:02 _____________M**_**__________L___Y_______A______________L_P_D____________RT__EL_T_**_**____QLEL_TT

DQB1*03:04 ____________AM**_**__________Y___Y_______A______E_______L_P_A____________RT__EL_T_**_**____QLEL_TT

DQB1*03:05:01 _____________M**_**______________Y_______A______________L_P_A____________RT__EL_T_**_**____QLEL_TT

DQB1*03:09 ____________AM**_**__________Y___Y_______A______E_______L_P_D____________RT__EL_T_**_**____QLEL_TT

DQB1*03:19 ____________AM**_**__________Y___Y_______A______E_______L_P_D____________RT__EL_T_**_**____QLEL_TT

DQB1*0402 ________F____M**_**______________Y_______A______________L__LD________DI__ED_____T_**_**____QLEL_TT

DQB1*06:01:01 __P_____L___AM**_**__________Y___Y______D___________________D________DI__RT__EL_T_**_**_______F___

**DQB1*06:02** **________F____M___________L___Y_______A__________________D_____________T__EL_T_________F___**

DQB1*06:03 _____________M**_**__________L___________A__________________D_____________T__EL_T_**_**_______F___

DQB1*06:04:01 _____________M**_**__________L___________A_______________________________RT__EL_T_**_**______G____

DQB1*06:09 _____________M**_**__________L___Y_______A_______________________________RT__EL_T_**_**______G____

**9 6 4 | ~4 4 6 9 ~ 7 @@@@@@@ 9 7 7 44 4 ~ 4|~!! 11 11**

**| + 7 ! - ~ ~ ! ! 7 - |+**

**| |_________________| - + ~ 6 |__||**

**| - |**

**|_______________________________S-S_____________________________|**

**91 100 110 120 130 140 150 160 170 180**

**DRB1*01:01 V______K__VY__K_QP_Q_____V___SG___GS_E______G___K______G__Q_______T_____TV_RSGE____Q_____V**

DQB1*05:01:01 LQRRVEPTVTISPSRTEALNHHNLLI**C**SVTDFYPSQIKVRWFRNDQEETAGVVSTPLIRNGDWTFQILVMLEMTPQ**RGD**VYT**C**HVEHPSL

**DQB1*05:02:01 ___________________________________H______________________________________________________**

DQB1*05:03:01 __________________________**_**_______________________________________________________**_**_______

DQB1*05:04 __________________________**_**________H______________________________________________**_**_______

**DQB1*02:01:01 _________________________V________A_______________________________________________________**

**DQB1*02:02 _________________________V________A_________G_____________________________________________**

DQB1*03:01:01 _________________________V**_**_______A______________T__________________________H_____**_**_______

**DQB1*03:02:01 _________________________V________A______________T________________________________________**

DQB1*03:03:03 _________________________V**_**_______A______________T________________________________**_**_______

DQB1*03:04 _________________________V**_**_______A______________T__________________________H_____**_**_______

DQB1*030501 _________________________V**_**_______A______________T________________________________**_**_______

DQB1*03:09 _________________________V**_**_______A______________T__________________________HA-___**_**_______

DQB1*03:19 _________________________V**_**_______A______________T__________________________H_____**_**_______

DQB1*04:02 _________________________V**_**_______A______________T________________________________**_**_______

DQB1*06:01:01 _________________________V**_**_______G_________________________________________H_____**_**_______

**DQB1*06:02 _________________________V________G_______________________________________________________**

DQB1*06:03 _________________________V**_**_______G_______________________________________________**_**_______

DQB1*06:04:01 _________________________V**_**_______G____Q__________________________________________**_**_______

DQB1*06:09 _________________________V**_**_______G____Q__________________________________________**_**_______

**# ## # | /////////////// # RGD |**

**| ## |**

**|________________________S-S____________________________|**

**181 190 200 210 220 230 237**

**DRB1*01:01 T__L______R___________________L___A__F_YF_NQ__HSGLQPTGF_S**

DQB1*05:01:01 QSPITVEWRAQSESAQSKMLSGVGGFVLGLIFLGLGLIIRQRSRKG--------LLH

**DQB1*05:02:01** ______________________________________________--------___

DQB1*05:03:01 ______________________________________________PQGPPPAG___

DQB1*05:04 ______________________________________________--------___

**DQB1*02:01:01 ______________________I________________HH__Q__--------___**

**DQB1*02:02 ______________________I________________HH__Q__--------___**

DQB1*03:01:01 _N____________________I________________HH__Q__--------___

**DQB1*03:02:01 _N__I_________________I________________HH__Q__--------___**

DQB1*03:03:03 _N__I_________________I________________HH__Q__--------___

DQB1*03:04 _N____________________I________________HH__Q__--------___

DQB1*03:05:01 _N__I_________________I________________HH__Q__--------___

DQB1*03:09 _N____________________I________________HH__Q__--------___

DQB1*03:19 _N__I_________________I________________HH__Q__--------___

DQB1*04:02 _N__I_________________I________________HH__Q__--------___

DQB1*06:01:01 ________________N_____I____________________Q__PQGPPPAG___

**DQB1*06:02** ___________________________________________Q__--------___

DQB1*06:03 ___________________________________________Q__--------___

DQB1*06:04:01 ___________________________________________Q__--------___

DQB1*06:09 ___________________________________________Q__--------___

**🡨--trans-membrane--🡪**

Notations and Conventions: As in Supplementary Figure S1. Additional symbols are:

For the putative homodimerization patch of β49-55 residues are marked with @ at the bottom of the sequence and highlighted in light green. Homodimerization of HLA-DQ has never been observed in any of the respective crystal structures. Yet, F(ab΄)_2_ fragment of anti-HLA-DQ antibodies differentially ligate HLA-DQ molecules on the surface of human monocytes and lead to differential (compared to HLA-DR and –DP) downstream signal transduction^70^.The β167-169 RGD sequence (where it exists), verified by crystallography in five HLA-DQ molecules encoded in *cis* (several in multiple determinations) and one encoded in *trans*, is highlighted in olive.

1 Matsuoka, T., Tabata, H. & Matsushita, S. Monocytes are differentially activated through HLA-DR, -DQ, and -DP molecules via mitogen-activated protein kinases. *J Immunol* **166**, 2202-2208, doi:10.4049/jimmunol.166.4.2202 (2001).
