# Supplementary figures and images for: Nine residues in HLA-DQ molecules determine with susceptibility and resistance to type 1 diabetes among young children in Sweden
Source: Sci Rep. 2021 Apr 23;11:8821. doi: 10.1038/s41598-021-86229-8 (PMC8065060; doi:10.1038/s41598-021-86229-8)

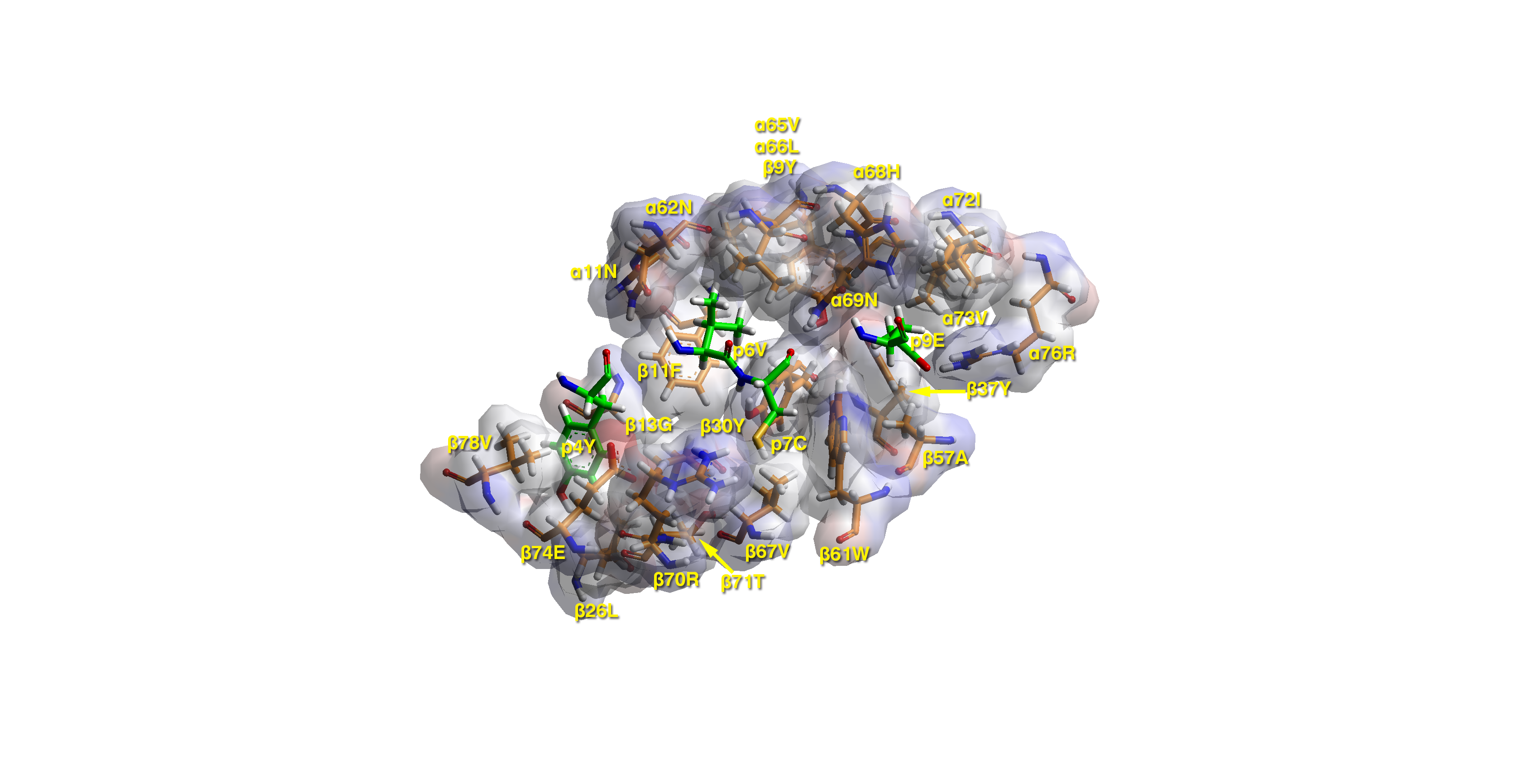

Supplement: Supplementary file 5 — Supplementary Information 5. [file 41598_2021_86229_MOESM5_ESM.tiff]

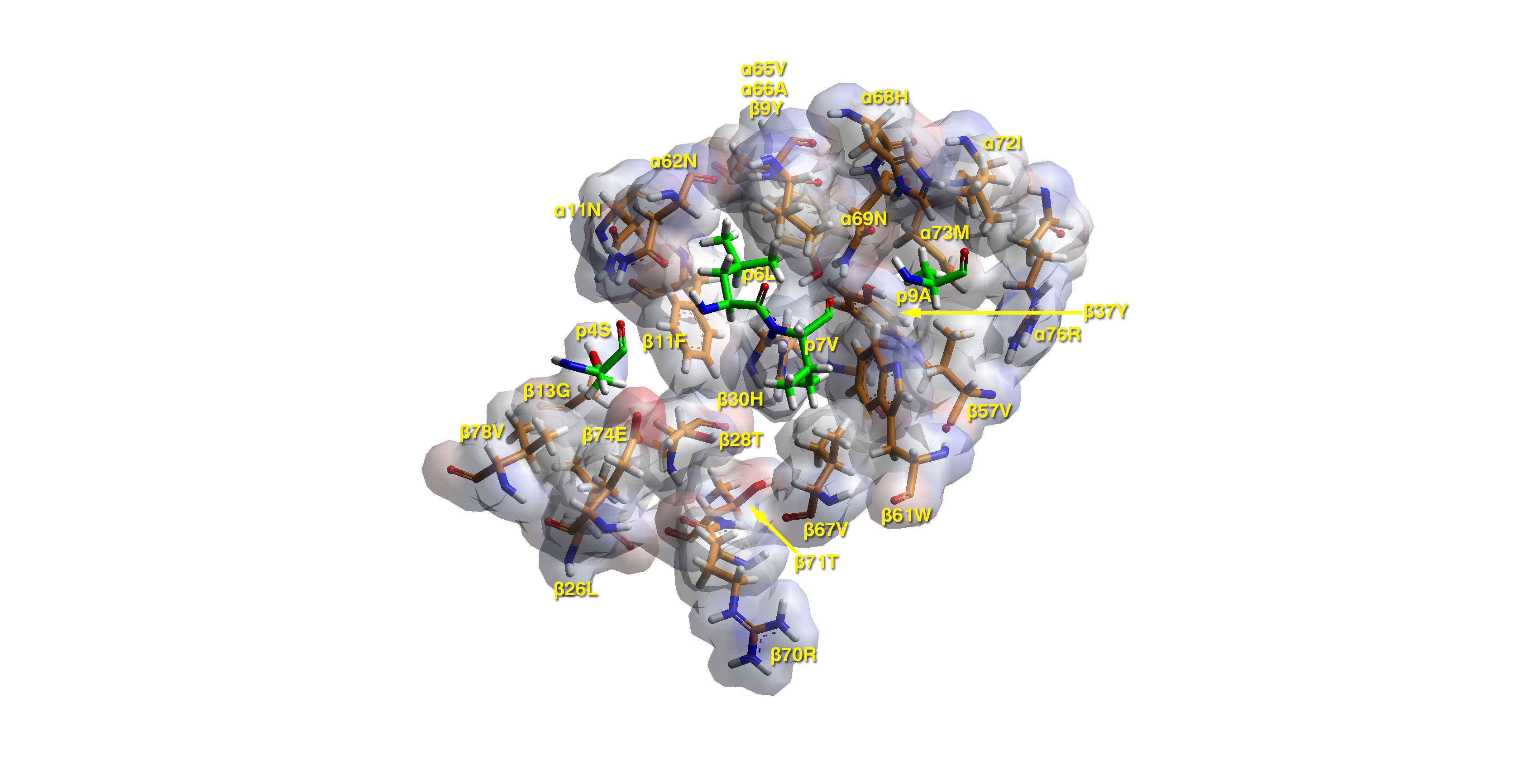

Supplement: Supplementary file 6 — Supplementary Information 6. [file 41598_2021_86229_MOESM6_ESM.tiff]

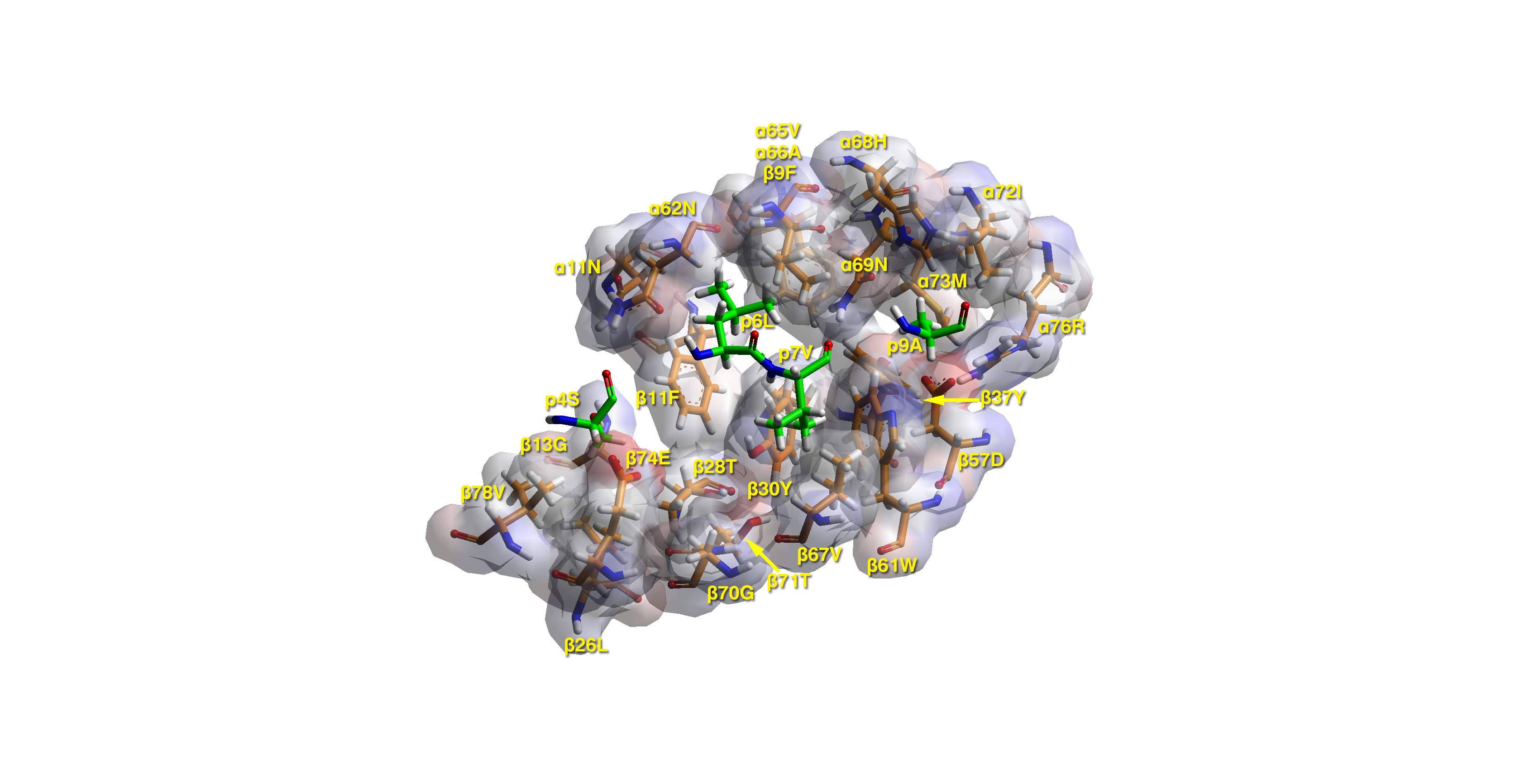

Supplement: Supplementary file 7 — Supplementary Information 7. [file 41598_2021_86229_MOESM7_ESM.tiff]
